# Risk factors for return to work in workers who experienced cancer: a systematic review with meta-analysis

**DOI:** 10.3389/or.2026.1759182

**Published:** 2026-06-16

**Authors:** M. Franco, F. Buscema, A. Catalano, L. Dansero, L. Milani, G. W. Gilcrease, F. Ricceri

**Affiliations:** 1 Centre for Biostatistics, Epidemiology and Public Health, Department of Clinical and Biological Sciences, University of Turin, Turin, Italy; 2 Department of Translational Medicine, University of Piemonte Orientale, Novara, Italy

**Keywords:** cancer, meta-analysis, meta-regression, neoplasm, return to work, risk factors, RTW, workers

## Abstract

**Background:**

The number of people who have experienced cancer is increasing, and by 2040 cases are expected to rise by 40%, with a high prevalence among working-age individuals. Many people who experienced cancer (pwEC) face reduced work ability due to health, personal, and occupational factors. Therefore, this study aims to identify the main factors that hinder RTW among pwEC.

**Methods:**

A systematic literature search was conducted via PubMed, CINAHL, and the Cochrane Library, covering studies published between January 2012 and June 2024. Inclusion criteria focused on studies analysing RTW and Time to RTW in relation to sociodemographic, clinical, and occupational factors. Meta-analysis grouped studies by design, exposure, and effect measures. Fixed or random effects models were applied based on heterogeneity. Risk of bias was assessed using NOS and RoB2 tools. Certainty of evidence was assessed using GRADE tool and STROBE checklist.

**Results:**

Among 2,151 screened articles, 43 met the inclusion criteria. Chemotherapy (OR = 0.56, 95% CI: 0.52–0.60) and radiotherapy were associated with a reduced likelihood of RTW. Women were more likely than men to RTW. Manual labour was linked to a lower likelihood of re-employment than white-collar jobs (OR = 0.84, 95% CI: 0.78–0.89). In terms of timing, male sex and chemotherapy delayed the process, whereas radiotherapy accelerated it (HR = 1.03, 95% CI: 1.00–1.06).

**Conclusion:**

Identifying barriers to RTW after cancer is essential for improving quality of life and social reintegration in people who have experienced cancer. These findings highlight the complexity of RTW and the need for a personalized approach. However, the evidence is limited by heterogeneity and differences in study design.

**Trial registration:**

The protocol was registered in PROSPERO (registration number: CRD42022384364).

## Rationale

Although the literature refers to people who have overcome a cancer diagnosis as survivors, the term “cancer survivor” is increasingly considered stigmatising and outdated. This shift reflects the growing number of individuals who fully recover from the disease. As a result, the more inclusive term “people who experienced cancer” (pwEC) is now preferred (1). The number of pwEC is rapidly increasing, according to the World Health Organization (WHO) (2). The relatively stable incidence, increase in screening programs, improvement in drug efficacy, and advanced treatment techniques have enhanced disease management and improved prognosis (3). The long-term life expectancy of pwEC is increasing in most developed countries (4). Moreover, in high-income countries, the 5-year relative survival for all cancers ranges between 46.9% in England and 68.0% in the United States, with an overall average of 54% (4). Specifically, individuals of working age account for nearly 50% of new diagnoses and more than 1/3 of pwEC (5). However, the Global Cancer Observatory estimates that the number of new diagnoses will increase by approximately 40% by 2040 compared with the incidence in 2020 and that new diagnoses will increasingly affect working-age people (6). Moreover, the retirement age has risen in many countries, contributing to the growing number of employable people (including pwEC) (5). Several studies have reported that return to work (RTW) for pwEC is crucial for both individuals and society. For individuals, it may contribute to an increase in their quality of life and perceived wellbeing (7). Indeed, resuming work may give back the feeling of returning to normality and playing an active role within the community. It also allows them to recover a good financial position after an uncertain treatment period (8). From a societal point of view, a cost analysis across the European Union estimated that 60% of the economic burden of cancer was incurred in non-healthcare areas, with almost €43 billion in lost productivity attributable to early death and €9.43 billion in lost working days due to cancer-related morbidity (5, 9). In pwEC who return to work, their ability to work is often impaired by cancer-related symptoms. Fatigue, pain, surgery and chemotherapy outcomes, recurrence, and cognitive and physical complications may occur, leading to a reduction in working hours or a greater need for sick leave (10). In addition, difficulties may also develop within the work context that hinder the return to work of individuals undergoing treatment for cancer illness: lack of support from colleagues, lack of work adjustment or lack of flexibility from the employer are the barriers most frequently reported in several qualitative studies by individuals who have already returned to work (11). The ICF model developed by the WHO (12) describes work as a crucial aspect of an individual’s social participation, highlighting health conditions, environmental factors, and personal factors related to workforce involvement. This perspective is particularly relevant in the context of oncology, where a person’s ability to work is influenced both by the effects of the disease and by broader psychosocial and work-related factors. For this reason, in line with the ICF conceptual framework, this review focused on the clinical, sociodemographic, psychological and occupational determinants of return to work following a cancer diagnosis. While several systematic reviews (13–17) have addressed the issue of return to work among individuals with a history of cancer, none have concurrently considered the full spectrum of cancer types together with sociodemographic, clinical, psychological, and work-related exposures, nor have they systematically compared the relative impact of these factors on RTW outcomes. Moreover, existing reviews have focused largely on European populations, with limited consideration of variability across different global contexts. Understanding the implications of cancer for social outcomes is necessary to enable decision-makers to identify the best strategy to address the burden of return to work that pwEC face. In the literature, few studies have explored the clinical, psychological, sociodemographic, and occupational factors that may hinder the RTW in workers with cancer.

## Objectives

This study aims to identify recent evidence on the sociodemographic, clinical, psychological, and occupational barriers to returning to work for people who have experienced cancer.

## Methods

We followed the Preferred Reporting Items for Systematic Reviews and Meta-Analyses (PRISMA) guidelines to conduct this systematic review (18). The protocol was registered in PROSPERO (registration number: CRD42022384364).

### Eligibility criteria

We included all Randomized Control Trials (RCTs) and longitudinal and cross-sectional studies published in English since 2012 that focused on adults aged 18 to 65 who had received a cancer diagnosis and were in paid work at the time of diagnosis. With respect to exposure, we considered all therapeutic interventions recommended by healthcare protocols for the treatment and care of individuals diagnosed with cancer, sociodemographic characteristics, work-related conditions, and psychological factors that may influence the RTW process. To be included, the studies had to collect information on return to work at least within 6 months and no later than 2 years after diagnosis. Data on return to work could be collected via follow-up interviews or questionnaires. For our study, we considered both dichotomous data of occurrence at the event (return to work: yes or no) and time factor data (days/months until return to work). Studies in which population characteristics were not collected or studies in which population characteristics were not stratified for RTW events, were excluded. We excluded qualitative, case report, case series, reviews and systematic reviews. Conference abstracts and grey literature were not considered.

### Information sources and search strategy

We searched the following electronic databases, PubMed, CINAHL, and the Cochrane Library Database, from January 2012 to June 2024 and used Boolean operators and MeSH terms to include term variations. Table 1 reports the search strategy used for each database. We used “Unemployed” and “unemployment” to include people who lost their jobs due to cancer. Missing information was retrieved by contacting the authors.

**TABLE 1:** Search strategy by database.

Database	Query strings	Results
PubMed	1. Neoplasm/2. Neoplasm* or cancer* or tumor*.ti,ab 3. Survivor* or experience.ti,ab 4. or/1–3 5. Return to work/6. ‘return to work’ or rtw or ‘work resumption’ or ‘back to work’ or ‘work re-entry’.ti,ab 7. Unemployment/ 8. Unemploy*.ti,ab 9. or/5–8 10. 4 and 9 11. Limit 10 to adults (18+) 12. Limit 10 to pubblication year: 2012 to 2024 13. Limit 10 to English language	1,270
CINAHL	1. Neoplasms/2. Neoplasm* or oncology or cancer or tumor or malignancy.ti,ab 3. Survivor* or experience. ti,ab 4. or/1–3 5. ‘return to work’ or ‘back to work’ or ‘job re-entry’ or ‘work resumption’ or rtw or ‘work re-entry’.ti,ab 6. Unemploy*.ti,ab 7. 5 or 6 8. 4 and 7 9. Limit 10 to adults (18–65) 10. Limit 10 to pubblication year: 2012 to 2024 11. Limit 10 to English language	503
Cochrane library	1. Neoplasms/2. Cancer Survivors/3. Cancer or cancer survivor*.ti,ab,kw 4. or/1–3 5. Return to work/6. ‘return to work’ or ‘back-to-work’ or rtw or job re-entry.ti,ab,kw 7. Unemployment/ 8. (unemploy*).ti,ab,kw 9. or/5–8 10. 4 and 9 11. Limit 10 to pubblication year: 2012 to 2024 12. Limit 10 to English language	378

Ti, title; ab, abstract; kw: keywords.

*: It is used to search for all words that begin with that root.

### Selection, data collection process, and data items

We performed article selection in two steps. First, after removing duplicates, we selected titles and abstracts that met the eligibility criteria; two authors (MF and AC) screened the studies independently. At the end of this session, the studies selected by the two authors were compared. In cases of disagreement, a third author (FR) was consulted. In the second step, two authors (MF and AC) screened the full texts of the previously selected studies. As before, we compared the final selection and discussed it. We resolved any disagreements by consulting a third author (FR). Zotero© was used to manage the selection. If the full-text of potentially eligible articles was unavailable or did not include data relevant to our analysis, the corresponding author was contacted for each article to request the data. The primary outcomes that we considered were RTW (yes or no) and Time to RTW. The secondary outcomes were current employment status and changes in the work environment. If the study design was an RCT, the outcomes of interest were related to the gap between the control group and intervention group to detect whether the intervention (or not an intervention) could favour the return to work. We extracted the outcomes from the Results and Findings sections of the article to prevent the authors’ opinions from influencing.

### Risk of bias assessment and quality of evidence

We assessed the risk of bias of the included observational studies via Newcastle-Ottawa Scale (NOS) tool (19). The NOS evaluates each study across three main domains: selection of study groups, comparability of groups, and determination of exposure or outcome (depending on whether the study is a case-control or cohort study). Each item may be assigned one star if it meets the quality criteria, with a maximum of nine stars per study (one star for each item, except for comparability, which may receive two). Higher scores indicate a lower risk of bias. We used the following classification based on the total score: 7–9 stars = low risk, 4–6 = some concerns, 0–3 = high risk, as suggested in the literature (20). We used an adapted version of the tool for assessing the risk of bias in cross-sectional studies (21). To assess the risk of bias in RCTs, we used the revised Cochrane Risk of Bias tool for randomised trials (RoB2) (22). RoB2 is structured into a fixed set of domains of bias, focusing on different aspects of trial design, conduct, and reporting. Within each domain, a series of questions (“signalling questions”) aims to elicit information about features of the trial that are relevant to the risk of bias. An algorithm proposes a judgment about the risk of bias arising from each domain on the basis of answers to the signalling questions. Judgements can be “low” or “high” risk of bias or can express “some concerns”. The Grading of Recommendations, Assessment, Development, and Evaluations (GRADE) (23) and the STrengthening the Reporting of OBservational studies in Epidemiology (STROBE) checklist (24) was used to assess the quality of evidence. Two authors (MF, FB) applied each of the above tools independently. Any discrepancies that emerged in the process of evaluating the studies were resolved by consulting a third author (FR).

### Exposures

Concerning sociodemographic factors, we collected data on age, gender, education, cohabitation (in some cases, using marital status as a proxy), ethnicity, and income. For clinical factors, we collected information about the tumours’ clinical stage, comorbidities, chemotherapy, radiotherapy, hormone therapy, and surgery. Concerning occupational factors, we collected information about the type ofjob/contract (full-time, part-time, self-employed) and the type of occupation (blue-collar, white-collar). With respect to psychological factors, we collected data about depression and anxiety. We set the independent variables as follow: aged 50 or more versus aged up to 49, male versus female, having a high school diploma or more versus not, cohabiting versus not, having a medium-high income versus low income, having a clinical stage of II or more versus stage up to I, having a comorbidity versus not, undergoing chemotherapy versus not, undergoing radiotherapy versus not, undergoing surgery versus not, having a part-time contract versus having a full-time contract, being self-employed versus being an employee, and having a blue-collar job versus a white-collar job. These cut-offs were defined based on the most reported categorizations across the included studies in order to ensure comparability and maximise the number of studies eligible for meta-analysis. In particular, for age, the ≥50 versus <50 years threshold reflected the most frequently used grouping in primary studies. For educational level and income, higher categories were grouped to allow comparison with the lowest category, with the aim of better capturing potential socioeconomic disparities in outcomes. As anticipated, we conducted meta-analyses on each exposure (factor) for each outcome (return to work, time to return to work).

### Effect measures and synthesis methods

For observational studies, we planned to synthesize the results of the dichotomous outcomes (return to work) with a meta-analysis model using the Odds Ratio (OR). We decided to use the adjusted ORs and 95% Confidence Intervals (CIs) reported in the study results section. If the studies with the outcome “return to work” reported no odds ratios in the results, we calculated crude odds ratios by constructing contingency tables using the frequencies reported in the population descriptive tables. For the outcome “time to return to work” as a time-to-event outcome, representing the relative rate of returning to work overtime between comparison groups, we used the Hazard Ratio reported in the Findings section for every record. Given the large number of factors explored, we decided to list the studies according to the three macro categories of exposure. In addition, we reported which outcome of interest each study investigated. Next, we grouped the studies according to the availability of data for each exposure factor and applied meta-analysis models for every single factor for both outcomes, returning to work and the time to return to work. To estimate the OR of the outcome “return to work”, we defined “Yes” as the response variable. We applied the random effects model because of the high variability of the factor types and their effects on outcomes. Weights were assigned via the Mantel‒Haenszel method for the OR and the inverse-variance method for the HR. Between-study heterogeneity is expected and reflects real-world variability in clinical pathways and labor market conditions. Therefore, the random-effects framework was considered appropriate not only as a statistical adjustment, but as a conceptual model acknowledging that effect sizes may legitimately vary across settings. We calculated a random-effects 95% prediction interval for meta-analyses with at least three studies to synthesize the effects of various exposures on the outcome. Given the anticipated heterogeneity, pooled estimates were interpreted in conjunction with 95% prediction intervals, which provide an estimate of the range of effects that may be expected in future populations. This approach allows for a more clinically meaningful interpretation of the results, particularly when the magnitude and direction of associations may differ across contexts. We assessed the impact of between-study heterogeneity by inspecting the forest plots and calculating the tau-squared and the I-squared statistics, respectively. The 95% CIs around the tau-squared and the I-squared values were calculated to evaluate confidence in these metrics. We adopted the I-squared thresholds of >75% to be considered signs of considerable heterogeneity, but we also judged the evidence for this heterogeneity (through the 95%CI) and the localization on the forest plot. Moreover, we conducted subgroup and meta-regression analyses on those studies where the results revealed a directional change in risk over the years to evaluate whether and what factors may have led to this shift. To investigate the presence of publication bias, we used funnel plots and Egger’s test. All analyses were run in STATA 18.0 by one author (FB).

### Analysis of heterogeneity and publication bias

Heterogeneity was evaluated via the I^2^ statistic. Fixed effects and random effects models were selected on the basis of study design and data characteristics, with the magnitude of I^2^ used to support model appropriateness. Meta-regression analyses were conducted for exposures presenting high heterogeneity to explore potential sources of variability and better understand the underlying causes of the observed heterogeneity. To assess potential small-study effects, funnel plots, Egger’s regression test (25), and the trim-and-fill method (26) were employed. The trim-and-fill method was applied to asymmetrical funnel plots to estimate and adjust for potentially missing studies through an iterative procedure based on a linear estimator.

## Sensitivity analyses

In addition, we conducted a sensitivity analysis regarding sex exposure, excluding from the analysis studies that focused on sex-specific cancers (breast, uterine, ovarian, cervical, prostate, testicular) or those studies that examined different types of cancer but did not differentiate between sex-specific cancers in their analyses or descriptive data. Moreover, we conducted a sensitivity analysis by excluding studies with a high risk of bias or a lower certainty of evidence.

## Results

### Study identification and selection

**FIGURE 1:**
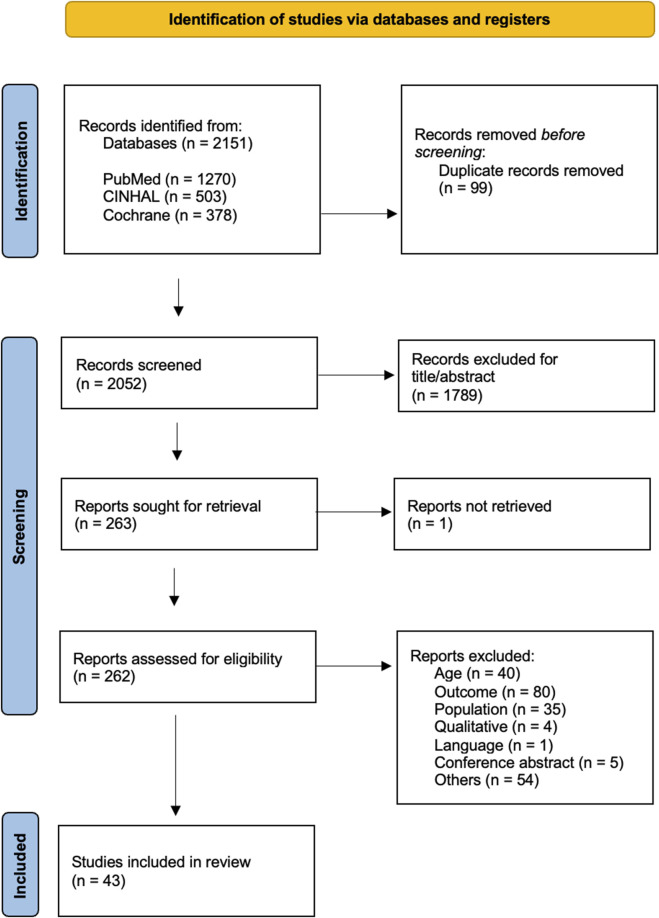
PRISMA 2020 flow diagram for new systematic reviews that included searches of databases and registers only.

The initial search pool included 2,151 articles extracted from three different electronic databases: PubMed (1,270), the Cochrane Library (378), and CINAHL (503). After duplicate removal (n = 99), 1789 records were excluded for title and abstract. The full texts of the remaining 263 articles were subsequently assessed following the same eligibility criteria previously specified. One of these potentially eligible records was not retrieved, and 9 records did not report the appropriate effect measures for the synthesis regarding some exposure. After requesting the data from the corresponding authors via email, we received three satisfactory responses (27–29). At the end of the process, 43 records were included in the review (27–69) (Figure 1). Table 2 includes information about the author, tumor location, publication year, year of recruitment, study design, country, sociodemographic, clinical, and occupational factors, and outcomes collected for each included study. Excluded studies and the reasons for their exclusion are detailed in the Supplementary Materials S1.

**TABLE 2:** Information about the data extracted from the included studies.

Study information								
Author	Tumor analysed	Year of recruitment	Study design	Country	Sociodemographic factors	Clinical factors	Occupational factors	Outcome
Arndt (2019)	- Breast - Colorectal - Prostate	2009–2011	Cohort	Germany	- Sex - Cohabiting	Stage	N/A	RTW
Basalathullah (2021)	Brain	2018–2020	Cohort	India	- Age - Sex - Education	Stage	- Type of contract - Designation of workers	RTW
Bennett (2018)	Prostate	2015–2017	Cohort	United Kingdom	- Age - Cohabiting	- Stage - Comorbidities - Radiotherapy	Type of contract	RTW
Blinder (2012)	Breast	2004–2005	Cohort	United States	- Cohabiting - Education	- Comorbidity - Radiotherapy - Chemotherapy	N/A	RTW
Caumette (2021)	Breast	2012–2017	Cohort	France	- Age - Cohabiting	- Stage - Comorbidity - Radiotherapy - Chemotherapy - Surgery	N/A	RTW
Check (2019)	Oropharyngeal	2000–2013	Cross- sectional	United States	- Age - Sex - Education	- Stage - Radiotherapy - Chemotherapy	N/A	RTW
Chen S (2019)	Oral	2015–2017	Cross- sectional	Taiwan	- Age - Cohabiting - Education - Income	- Stage - Radiotherapy - Chemotherapy - Surgery	Designation of workers	RTW
Chen W (2021)	Not specified	2004–2010	Cohort	Taiwan	- Age - Sex	- Stage - Comorbidities - Radiotherapy - Chemotherapy - Surgery	- Type of contract - Type of occupation	Time to RTW
Chen Y (2020)	Oral	2004–2015	Cohort	Taiwan	- Sex - Income	- Stage - Radiotherapy - Chemotherapy - Surgery	Designation of workers	Time to RTW
Chiu (2023)	Head and neck	2018–2021	Cross- sectional	Taiwan	- Sex - Education	- Stage - Radiotherapy - Chemotherapy	N/A	RTW
Colombino (2020)	Breast	2012–2014	Cross- sectional	Brazil	- Age - Education - Income	- Stage - Radiotherapy - Chemotherapy	Designation of workers	RTW
Dahl (2014)	Prostate	2008–2009	Cohort	Norway	- Age - Education	N/A	Type of contract	RTW
den Bakker (2020)	Colorectal	2012–2014	Cohort	Netherlands	- Age - Sex	N/A	Type of contract	RTW
Dumas (2020)	Breast	2012–2015	Cohort	France	- Age - Cohabiting - Income	- Stage - Radiotherapy - Chemotherapy - Surgery	- Type of contract - Designation of workers	RTW
Endo (2016)	Not specified	2000–2011	Cohort	Japan	Age	N/A	N/A	Time to RTW
Fiabane (2024)	Breast	2017–2019	Cohort	Italy	N/A	- Chemotherapy - Radiotherapy	- Designation of workers - Type of contract - Type of occupation	RTW
Gordon (2014)	Colorectal	2010–2011	Cohort	Australia	Sex	N/A	N/A	- RTW - Time to RTW
Hedayati (2013)	Breast	2006–2009	Cohort	Sweden	- Cohabiting - Education	Chemotherapy	N/A	RTW
Horsboel (2015)	Hematological	2011–2012	Cohort	Denmark	Sex	Comorbidity	N/A	RTW
Horsboel (2013)	Hematological	2000–2007	Cohort	Denmark	- Age - Sex - Education - Marital status - Income - Ethnicity	Comorbidity	N/A	Time to RTW
Ito (2015)	Not specified	2011–2011	Cross- sectional	Japan	- Age - Sex	- Radiotherapy - Chemotherapy - Surgery	Type of contract	RTW
Johnsson (2023)	Breast	1997–2011	Cohort	Sweden	- Age - Cohabiting	N/A	N/A	RTW
Kang (2022)	Not specified	2017–2018	Cross- sectional	South Korea	- Sex - Education - Income	Stage	Designation of workers	RTW
Landeiro (2018)	Breast	2012–2014	Cohort	Brazil	- Age - Education	- Radiotherapy - Chemotherapy - Surgery	N/A	RTW
Li (2021)	Breast	2012–2018	Cohort	China	- Age - Education	- Stage - Comorbidity - Radiotherapy - Chemotherapy - Surgery	Designation of workers	RTW Time to RTW
Lindbohm (2014)	Breast	1997–2002	Cross- sectional	North Europe (Denmark, Norway, Sweden, Iceland)	N/A	N/A	Designation of workers	RTW
Morales (2019)	Oropharyngeal	2018–2018	Cross- sectional	Australia	N/A	- Stage - Chemotherapy - Surgery	- Type of contract - Designation of workers	RTW
Paltrinieri (2020)	Not specified	2012	Cross- sectional	Italy	- Age - Sex - Cohabiting - Education	- Radiotherapy - Chemotherapy	Type of contract	RTW
Rashid (2021)	Lung	2015–2016	Cross- sectional	Germany	- Age - Sex - Income	- Stage - Comorbidity - Radiotherapy	- Type of contract - Designation of workers	RTW
Rosbjerg (2020)	Not specified	2016–2018	Cohort	Denmark	- Sex - Education	N/A	N/A	Time to RTW
Ross (2012)	Not specified/Breast^1^	N/A	Cross- sectional	Denmark	- Age - Sex - Cohabiting - Education	Stage	N/A	RTW
Rydén (2020)	Brain	2005–2015	Cohort	Sweden	- Age - Sex	Surgery	N/A	RTW
			(Matched)					
Schmidt (2019)	Breast	2010–2013	Cohort	Germany	- Cohabiting - Education	- Stage - Radiotherapy - Chemotherapy - Surgery	N/A	RTW
Sesto (2022)	Breast	2014–2015	Cross- sectional	United States	- Education - Income	N/A	Type of contract	RTW
So (2019)	Nasopharyngeal	2015–2016	Cross- sectional	Canada	- Age - Sex - Cohabiting - Income	Stage	Designation of workers	RTW
Starnoni (2018)	Brain	2012–2015	Cohort	France	Sex	Surgery	- Type of contract - Designation of workers	RTW
Tamminga (2019)	Not specified	2009–2010	RCT	Netherlands	Education	N/A	Chemotherapy	Time to RTW
Ullrich (2017)	Prostate	2010–2012	Cohort	Germany	Age	N/A	N/A	RTW
Ullrich (2018)	Prostate	2010–2012	Cohort	Germany	- Age - Education - Income	- Stage - Comorbidity	- Type of contract - Designation of workers	RTW
Ullrich (2022)	Prostate	2010–2012	Cohort	Germany	- Cohabiting	N/A	N/A	RTW
van loon (2021)	Brain	2003–2016	Cross- sectional	Netherlands	- Sex - Cohabiting - Education	- Radiotherapy - Chemotherapy	N/A	RTW
Yang S (2021)	Liver	2004–2010	Cohort	Taiwan	- Sex - Income	- Stage - Radiotherapy - Chemotherapy - Surgery	Designation of workers	Time to RTW
Yang Z (2022)	Lung	2004–2015	Cohort	Taiwan	- Sex - Income	- Stage - Comorbidity - Radiotherapy - Chemotherapy - Surgery	Designation of workers	Time to RTW

1 Analyses were conducted both for several types of cancer and breast only.

### Study characteristics

Overall, the analysis included a total of 43 studies, consisting of 14 cross-sectional (29, 35, 36, 39, 40, 49, 51, 54–57, 59, 61, 62), 28 cohort studies (27, 28, 30–34, 36, 37, 41–48, 50, 52, 53, 58, 60, 63, 65–68, 68), and one RCT (64). The selected studies spanned from January 2012 to June 2024. In terms of geographical distribution, 23 studies were conducted in Europe (28–31, 34, 41–43, 45, 47, 48, 50, 54, 56–60, 63–67) (including Norway, the Netherlands, Sweden, Denmark, Italy, and France), one in the United Kingdom (32), 4 in North America (33, 35, 61, 62) (United States of America, Canada), 2 in South America (40, 52) (Brazil), 10 in Asia (36–39, 44, 49, 51, 53, 68, 69) (Taiwan, India, China, Japan, and South Korea), and 2 in Australia (46, 55). The average follow-up duration was 2 years. The classification of RTW was consistent across studies, with most categorizing it as either RTW or not. Only one study (55) distinguished it further, classifying the outcome based on whether the individual returned with the same number of hours or fewer. The risk of bias was evaluated using the NOS tool for both cross-sectional and cohort studies, the RoB2 tool for RCTs. Quality of evidence was evaluated using the GRADE criteria and the STROBE checklist (Supplementary Materials S2-S4). Only 6 out of 43 articles were identified as low quality on the basis of the NOS tool (27, 29, 30, 35, 49, 61).

### Results for RTW

**FIGURE 2:**
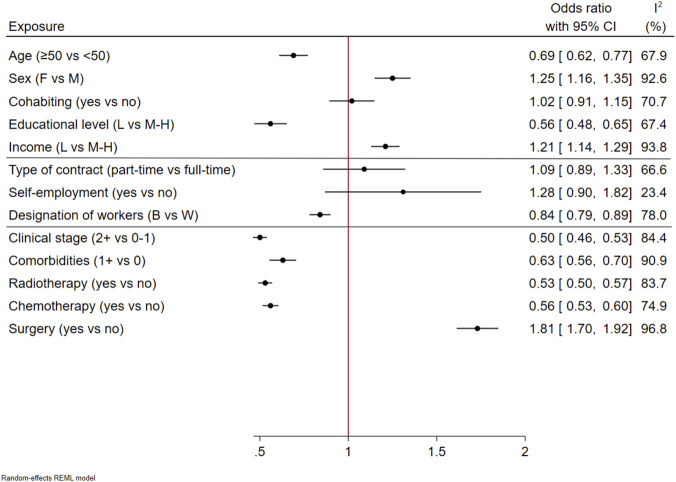
Summary of overall meta-analysis results on RTW outcome by exposure. I^2^, heterogeneity index. F, female; M, male. L: low level; M-H, medium-high level; B, blue-collar; W, white-collar.

The results of the meta-analysis are summarized in Figure 2. The individual meta-analyses conducted on each exposure are reported in the Supplementary Materials S5-S17. The analysis of sociodemographic factors revealed that being aged 50 years or older was associated with a reduction in the likelihood of returning to work (OR = 0.69, 95%CI: 0.62–0.77). In addition, low education levels were associated with a lower likelihood of returning to work (OR = 0.56, 95%CI: 0.48–0.65). Low income, on the other hand, is associated with a greater probability of RTW (OR = 1.21, 95%CI: 1.14–1.29). Moreover, women are more likely than men to return to work after having experienced cancer (OR = 1.25, 95%CI: 1.16–1.35). No statistically significant effect on the outcome was found for cohabiting (OR = 1.02, 95%CI: 0.91–1.15). For ethnicity, the information reported in the studies did not allow us to identify common categories for the studies concerned. Despite the consistency in the direction of several associations, substantial between-study heterogeneity was observed across most analyses (I^2^ values frequently exceeding 75%). This suggests that the magnitude of the associations varies across populations and contexts. Meta-regression analyses were therefore considered essential to explore potential sources of variability and to contextualize the pooled estimates.

In the assessment of clinical factors, a trend toward an increased risk of returning to work for patients undergoing surgery was found (OR = 1.81, 95%CI: 1.70–1.92). In addition, chemotherapy (OR = 0.56, 95%CI: 0.52–0.60) and radiotherapy (OR = 0.53, 95%CI: 0.50–0.57) also affected the outcome, suggesting that patients receiving such treatments have a reduced likelihood of returning to work. Finally, the analysis revealed that patients with comorbidities (defined as having at least one additional condition other than cancer) had a 37% lower chance of RTW than did those without comorbidities (OR = 0.63, 95%CI: 0.56–0.70). Even those with advanced cancer had a significantly greater risk of not returning to work (OR = 0.50, 95%CI: 0.46–0.53). Finally, it was not possible to conduct a meta-analysis of psychological factors because the studies that collected the information on these factors used different and non-comparable measurement tools. However, those that evaluated aspects such as anxiety and depression aligned in showing that both factors negatively affect the RTW process, reducing the likelihood of successful reintegration. Among occupational factors, manual workers had a lower probability of returning to work after their cancer status than white collar workers did (OR = 0.84, 95%CI: 0.78–0.89). In contrast, being self-employed (OR = 1.28, 95%CI: 0.90–1.82) or having a part-time contract (OR = 1.09, 95% CI: 0.89–1.33) seems to increase the risk of returning to work, even if the result is not statistically significant.

### Results for time to RTW

**FIGURE 3:**
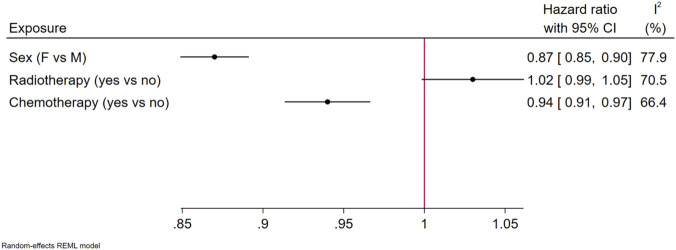
Summary of overall meta-analysis results on time to Return To Work outcome by exposure. I^2^, heterogeneity index; F, female; M, male.

Concerning Time to Return to Work, the data indicate that men take longer than women to RTW after having experienced cancer (HR = 0.87, 95%CI: 0.85–0.89). People who received chemotherapy (HR = 0.94, 95%CI: 0.92–0.97) tended to have a longer time to RTW. Those who underwent radiotherapy tended to show an increase in the rate of return to work over time (HR = 1.03, 95%CI: 1.00–1.06), albeit the result was at the threshold of statistical significance (Figure 3). It was not possible to synthesise the other types of exposure due to a lack of data. The individual meta-analyses conducted on each exposure are reported in the Supplementary Materials S18-S20.

### Meta-regression

**FIGURE 4:**
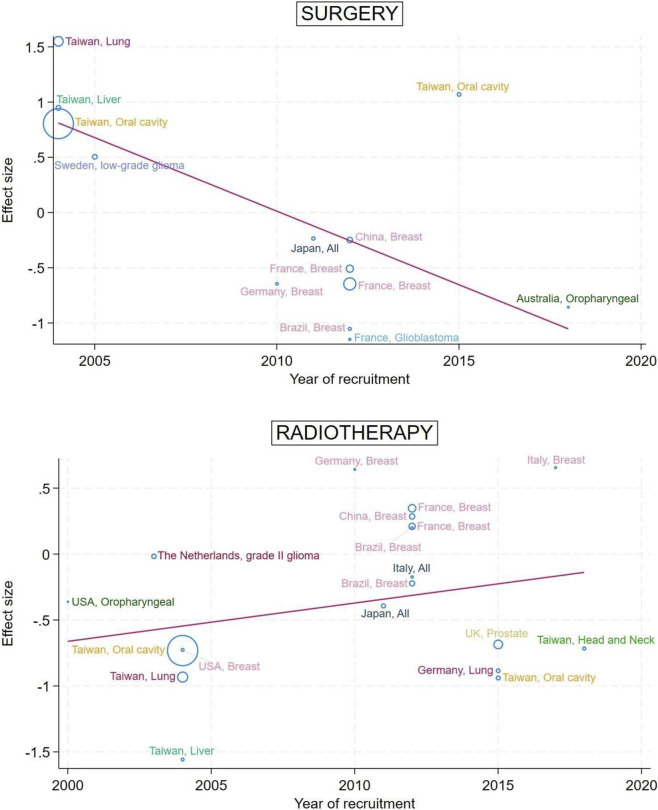
Meta-regression of the outcome Return To Work performed by surgery and radiotherapy exposure.

Due to a high level of heterogeneity, meta-regressions were also performed considering the exposure to surgery and radiotherapy, based on the year of recruitment of the studied population. This approach was adopted to explore the reasons for the high heterogeneity found in meta-analyses of these types of treatments. We performed meta-regressions with additional labels included for each study, such as country of publication and type of cancer analysed (Figure 4).

The meta-regression analyses revealed time-dependent patterns that provide important insights into the observed heterogeneity. For surgery, a clear negative trend was observed, indicating that more recent studies tend to report less favourable associations with RTW. This pattern is unlikely to reflect a negative effect of surgical techniques *per se*, but rather a shift in clinical practice over time. The results also showed that different types of cancer had different impacts on the likelihood of RTW (see breast cancer).

Conversely, radiotherapy showed a modest positive trend over time, suggesting an improvement in its association with RTW. This may reflect both technological advancements leading to reduced treatment-related morbidity and changes in clinical use, with radiotherapy increasingly integrated into less invasive or organ-preserving treatment strategies. Here, too, a significant effect of the type of cancer (breast) on the likelihood of RTW after radiotherapy was observed. Meta-regression analyses provided important insights into the sources of heterogeneity, highlighting that part of the variability in effect estimates was explained by cancer type and study context.

### Publication bias

A total of 39 studies analysed the associations between return to work and individual cancer history. Funnel plots were generated for each exposure examined in the meta-analyses, displaying the effect estimate using effect size and standard error as measures of precision. Visual inspection of these funnel plots indicated potential asymmetry, suggesting the presence of publication bias, which was further supported in some cases by the Egger test (P < 0.05). Both the random effects model and the Restricted Maximum Likelihood (REML) method were employed; specifically, REML was used to estimate heterogeneity among studies, improving the accuracy of the overall effect estimates in the presence of variations among the included studies. We chose to apply REML method because it is recommended when the number of studies included in each synthesis is relatively small (71). No significant changes in estimates were observed before and after adjusting for publication bias, indicating the robustness of the results and suggesting that any potential bias does not substantially impact the study’s conclusions (Supplementary Materials S21, S22).

### Risk of bias

**FIGURE 5:**
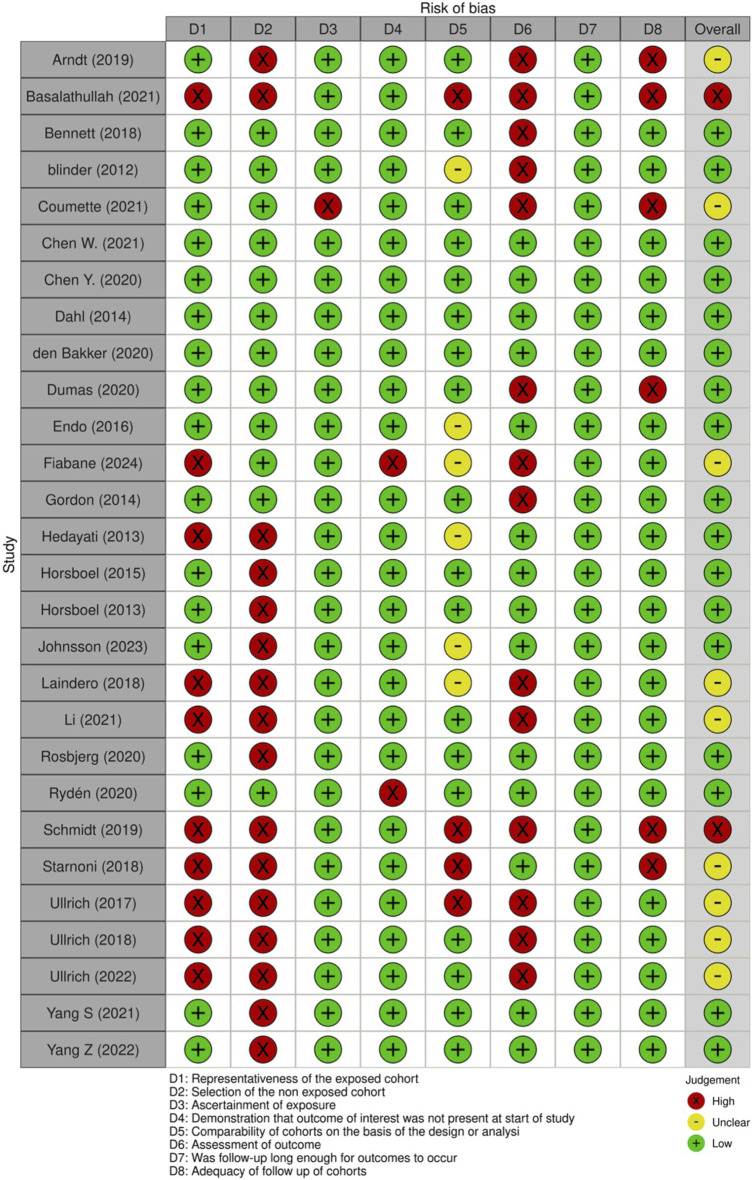
Risk of bias evaluations of cohort studies using Newcastle-Ottawa Scale.

**FIGURE 6:**
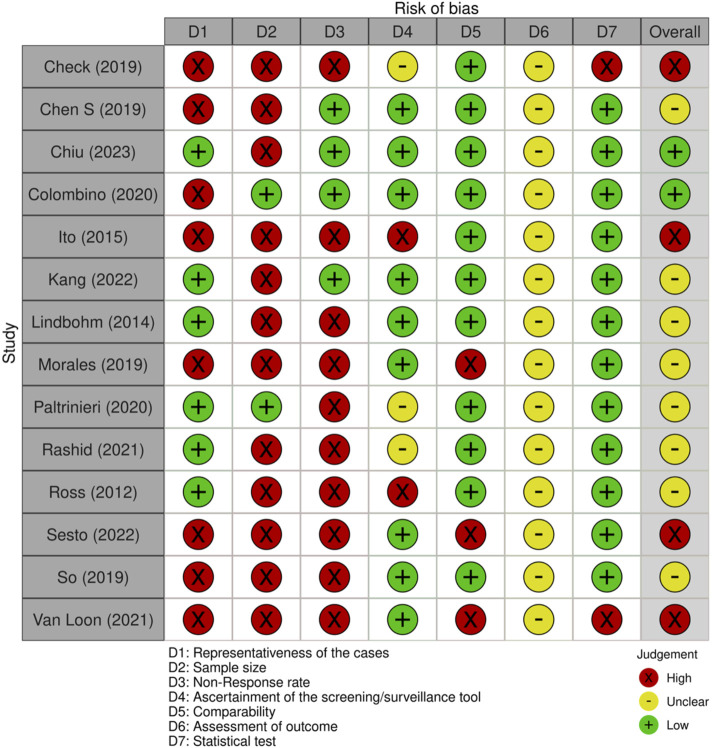
Risk of bias evaluation of cross-sectional studies using Newcastle-Ottawa scale (cross-sectional adaptation).

We use the Newcastle-Ottawa Scale (NOS) tool to assess risk of bias in cohort and cross-sectional studies and the RoB 2.0 tool to assess risk of bias in experimental studies. The results of NOS assessments were reported in Figure 5 and in Figure 6. Regarding observational studies, 6 of them showed a high risk of bias (27,29,30,35,49,61). The motivation for the evaluation is given in Supplementary Materials S37. The single experimental study included (64) showed a low risk of bias after assessment with RoB 2.0 tool (Supplementary Materials S3).

### Certainty of evidence

For all observational studies, evidence from cohort and case‒control studies was rated as “low”, “uncertain” or “high” risk of bias due to quality downgrading factors (under- or overmatching in case‒control studies, selection of exposed and unexposed in cohort studies from different populations, differences in measurement of exposure, differential surveillance for outcome in exposed and unexposed in cohort studies, failure to match for prognostic factors and/or adjustment in statistical analysis, failure of accurate measurement of all known prognostic factors, and incomplete or inadequately short follow-up). Most of the evidence was classified as low-grade risk (Table 3). The motivations of evaluations were reported in Supplementary Material S4.

**TABLE 3:** Certainty of evidence evaluations with Grading of Recommendations, Assessment, Development, and Evaluations (GRADE).

Year	Failure to develop and apply appropriate eligibility criteria (inclusion of control population)	Under- or over-matching in case-control studies	Selection of exposed and unexposed in cohort studies from different populations	Flawed measurement of both exposure and outcome	Differences in measurement of exposure (e.g., recall bias in case-control studies)	Differential surveillance for outcome in exposed and unexposed in cohort studies	Failure to adequately control confounding	Failure to match for prognostic factors and/or adjustment in statistical analysis	Failure of accurate measurement of all known prognostic factors	Incomplete or inadequately short follow-up	Overall results
First authors	​	​	​	​	​	​	​	​	​	​	​
Arndt (2019)	​	​	●	​	​	●	​	​	●^1^	●	●
Basalathullah (2021)	​	​	●^2^	​	​	●^3^	​	​	●^4^	●	●
Bennett (2018)	​	​	●	​	​	●	​	​	●^5^	●	●
Blinder (2012)	​	​	●^6^	​	​	●	​	​	●	●	●
Caumette (2021)	​	​	●	​	​	●	​	​	●	●	●
Chen W (2021)	​	​	●	​	​	●	​	​	●	●	●
Chen Y (2020)	​	​	●^7^	​	​	●	​	​	●	●^8^	●
Dahl (2014)	​	​	●	​	​	●	​	​	●	●^9^	●
den Bakker (2020)	​	​	●	​	​	●	​	​	●	●^10^	●
Dumas (2020)	​	​	●	​	​	●^11^	​	​	●	●^12^	●
Endo (2016)	​	​	●	​	​	●	​	​	●	●^13^	●
Fiabane (2024)	​	​	●	​	​	●	​	​	●	●^14^	●
Gordon (2014)	​	​	●	​	​	●	​	​	●	●^15^	●
Hedayati (2013)	​	​	●	​	​	●	​	​	●	●^16^	●
Horsboel (2015)	​	​	●	​	​	●	​	​	●	●^17^	●
Horsboel (2013)	​	​	●	​	​	●	​	​	●	●	●
Johnsson (2023)	​	​	●	​	​	●	​	​	●	●^18^	●
Landeiro (2018)	​	​	●	​	​	●	​	​	●^19^	●^20^	●
Li (2021)	​	​	●	​	​	●	​	​	●	●^21^	●
Rosbjerg (2020)	​	​	●	​	​	●	​	​	●	●^22^	●
Rydén (2020)	​	​	●	​	​	●	​	​	●	●^23^	●
Schmidt (2019)	​	​	●	​	​	●	​	​	●	●	●
Starnoni (2018)	​	●	​	​	●^24^	​	​	●^25^	​	●^26^	●
Ullrich (2017)	​	​	●	​	​	●	​	​	●^27^	●^28^	●
Ullrich (2018)	​	​	●	​	​	●	​	​	●	●^29^	●
Ullrich (2022)	​	​	●	​	​	●	​	​	●	●^30^	●
Yang SW (2021)	​	​	●	​	​	●	​	​	●	●	●
Yang ZY (2021)	​	​	●	​	​	●	​	​	●	●	●

Rick of bias- per study	
Low	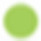
Uncertain	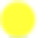
High	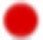

Combinations				
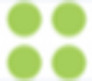	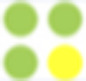	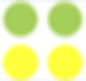	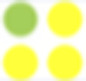	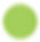
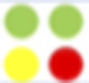	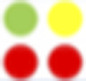	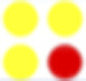	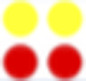	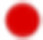

### Sensitivity analyses

Excluding studies that included sex-specific cancers in the synthesis between sex exposure and RTW outcome, we obtained a shift in the estimate towards null-value, resulting in a loss of statistical significance (Supplementary Materials S35). Regarding the synthesis between sex exposure and the outcome Time to RTW and the other sensitivity analyses the results remained consistent with the original results (Supplementary Materials S23-S34, S36), reinforcing the conclusion that the associations are robust and not dependent on lower-quality evidence.

## Discussion

This systematic review and meta-analysis aimed to explore what sociodemographic, clinical, psychological, and work-related factors may hinder the return to work of people who have been diagnosed with any type of cancer. The purpose of this study was to identify generalizable patterns of association between exposures and RTW outcomes rather than to estimate a single underlying “true” effect size, thus helping to identify population groups that may face greater difficulty in resuming work activities. The decision to pool estimates across heterogeneous contexts (including different cancer types, healthcare systems, and socio-economic environments) was aligned with the primary objective of this review. In this perspective, between-study heterogeneity is expected and reflects real-world variability in clinical pathways and labor market conditions. The high level of heterogeneity observed across analyses requires careful interpretation of pooled estimates. Rather than representing universally applicable effect sizes, these estimates should be interpreted as average associations across diverse contexts. In this framework, the results provide information on the direction and relative magnitude of associations, while acknowledging that the actual effect in a given setting may differ substantially. However, the results obtained appear to be largely aligned with the conclusions of other systematic reviews that analyse the topic of RTW for people who have experienced cancer. Moreover, beyond confirming previous findings, this study provides an additional contribution by integrating multiple exposure domains (sociodemographic, clinical, psychological, and occupational factors) across different cancer types within a comprehensive meta-analytic approach. This allows for a more integrated and cross-domain understanding of the determinants of RTW. Tavan et al. (72) reported similar results concerning the effects of chemotherapy, surgery, and radiotherapy on RTW. While these factors generally decrease the probability of returning to work, surgery has been found to improve the chances of work resumption. According to the systematic review by van Muijen et al. (73), individuals who underwent surgery as their only treatment had a greater probability of RTW than those who received combined therapies. Furthermore, less invasive surgery was associated with a favourable probability of RTW compared with invasive surgery. Given the publication period of the studies considered for exposure to surgery, the results obtained in our study may be due to the development of less invasive surgical techniques. The same conclusions can be drawn from meta-analyses conducted to assess the Hazard Ratio effect sizes of radiotherapy, although the association is small and of borderline statistical significance, and chemotherapy exposures. Concerning comorbidities, the results of our study align with those reported in the article by Forbes et al. (74). In both cases, the presence of one or more health conditions is a significant factor that may delay or hinder RTW. It is plausible that people facing not only cancer but also one or more morbidities may have greater difficulty recovering, or they may be more susceptible to the development of medical complications. In addition, Ota et al. reported significant similarities in clinical stage (75). As with the previous exposure, reliable statistical evidence was observed between having an advanced-stage cancer and the outcomes considered. Considering both age as a continuous and discrete variable, significant parallels emerged in the study by Handschel J. et al. (76), increasing age is associated with greater difficulties in RTW. Our primary results suggested that females had a greater probability than males of a successful RTW outcome. Similar results were reported in the study conducted by Argawal et al. (77). However, our sensitivity analysis showed a loss of significance with a clear shift towards the null value. Results were in line with those reported by Pascual et al. (78). This higher likelihood of RTW among women should therefore be interpreted cautiously, as it may be affected by unmeasured confounders such as cancer type, job characteristics, and working conditions. One possible explanation for this may be the inclusion of studies that collected information on sex-specific cancers, particularly breast cancer. Compared with those of other cancers, female breast cancer survival rates are increasing rapidly, and the RTW rate in this type of cancer is one of the highest (14). A study has shown that low income is strongly associated with RTW after a cancer diagnosis (79). Contrary to the findings reported by Wang L. et al. (79), which suggest a weaker relationship between income and work reintegration, evidence indicates that survivors with lower incomes are often compelled to return to work earlier to not suffer the burden of economic hardship, even when not fully recovered (8). This dynamic can be explained by nuanced factors. First, low-income workers often have limited savings or financial resources, making work reintegration an economic necessity rather than a choice. Furthermore, they are more likely to hold unstable jobs with fewer protections, which do not provide extended paid leave or greater flexibility to accommodate their recovery process (80). Economic pressures are often exacerbated by the lack of adequate health insurance or other forms of social support, forcing many individuals to return to work to cover medical expenses or meet their family’s daily needs. This situation increases the likelihood of premature re-entry into the workforce without completing physical and psychological recovery, with potential negative consequences for health and long-term job retention. Finally, most systematic reviews explore the relationship between higher levels of education and lower levels of education in relation to RTW. However, in our study, we opted to compare high/medium education levels with low education levels. The results we obtained are consistent with findings from similar research (77, 81). These findings could be explained by individuals with higher or intermediate educational attainment being more likely to occupy prominent positions and enjoy certain guarantees and protections than those with lower academic qualifications. Additionally, they are more often engaged in roles that are less physically demanding, which reduces their risk of injury. Notably, the analysis of self-employed workers, based on self-employment exposure, revealed a higher RTW rate compared to employed workers, even though this difference was not statistically significant in our study. Unlike our findings, some studies have observed that self-employed professionals are more likely to return to work, interpreting this data as being due to a lack of employment protections (82). A similar pattern was observed among part-time employees (82). Conversely, the findings of our study regarding worker roles align with those reported in several previous studies (76, 77). Similarly, our results indicate that managerial or white-collar roles are associated with fewer challenges in the RTW process, whereas manual or blue-collar occupations tend to correlate with greater difficulties. This trend may be attributed to the fact that roles involving higher levels of responsibility often provide greater job security and protection, which can mitigate the impact of an extended absence from work. Finally, the findings from the meta-regression analysis highlight that the type of treatment and the stage of the pathology interact in complex ways, significantly influencing the process of returning to work. These insights have important implications for designing personalized occupational reintegration strategies for cancer patients. For breast cancer, as noted by Keelan et al., surgical treatments have evolved from highly invasive procedures to less invasive approaches, often combined with radiotherapy (83). Meta-regression analysis suggested that, initially, surgery was widely used regardless of tumor severity, and patients who underwent surgical treatment were more likely to return to work. Over time, surgical selection criteria have become more restrictive, reserving surgery for more severe cases while favouring multimodal therapy regimens, including radiotherapy and hormonal therapies for less aggressive tumors (84). As a result, patients who underwent surgery more recently probably had advanced tumors that did not allow the use of less invasive alternatives, making their return to work less likely because of the greater severity of their condition. Moreover, advanced tumors often necessitate more aggressive and invasive surgical interventions, leading to physical and psychological complications (85) that can extend recovery time and hinder occupational reintegration. Radiotherapy is commonly employed for smaller tumors or as an adjuvant treatment after surgery to reduce the risk of recurrence (86). Compared with major surgical treatments or combination therapies such as chemotherapy, it typically results in fewer debilitating side effects, allowing for a faster recovery and a smoother RTW, as shown in the meta-regression analysis.

### Public health implications

This study’s global approach could have important impacts on public health. By examining the various factors that affect RTW outcomes for pwEC, the results could be used to develop targeted interventions to improve their reintegration into the workforce. These interventions could reduce long-term disability risks, support social inclusion, and help with economic recovery for a larger group of people. In addition, supporting RTW could lead to more efficient use of healthcare resources, as it may decrease the need for extended medical treatments and reduce pressure on healthcare systems. Encouraging successful RTW could also improve the mental wellbeing of cancer survivors, helping them feel more socially connected and improving their overall quality of life. On the other hand, providing financial support to people on low incomes could reduce the potential pressure to return to work in poorly health conditions, giving them the opportunity to invest more time and resources in their health. Finally, this research could help shape policies and workplace adjustments that create more supportive environments for pwEC, leading to a more balanced approach to cancer care that includes both medical treatment and psychological support.

### Implication for future research

Despite improvements in understanding the outcomes of RTW after cancer, some important gaps remain that require further investigation. First, our review highlighted difficulties with the availability and consistency of data on psychosocial determinants, particularly mental health and ethnicity. These factors are recognised in the literature as key predictors of RTW, but are often measured inconsistently or underestimated across studies, limiting comparability and synthesis. To address this issue, future research should prioritise the development and adoption of standardised measures for psychosocial variables. For example, the use of widely validated and comparable mental health assessment tools (such as the Hospital Anxiety and Depression Scale, the Patient Health Questionnaire, or other constructs in line with the International Classification of Functioning frameworks) would improve harmonisation across studies. Similarly, implementing social disadvantage using minority status rather than ethnic categories may allow for better capture of structural and contextual influences on RTW.

### Strengths and limitations

Importantly, our study has several limitations. First, some included studies gathered information retrospectively, which could introduce recall bias into the data. Additionally, the tendency of some studies to report only statistically significant results may heighten the risk of publication bias. Another limitation involves the differences in methods of data collection and variable categorization among the included studies, which could affect data comparability, as was the case for the analysis of ethnicity. Moreover, the combined use of crude and adjusted odds ratios in the analyses, without a clear distinction between the two, could lead to potential bias in the estimates of the syntheses, both because the crude effect measures could over- or underestimate the actual effect, and because the adjusted estimates controlled for different variables across studies, limiting both the comparability and accuracy of the estimates and raising the heterogeneity of the meta-analyses conducted. Furthermore, national differences in labor policies, discrepancies in the methods used to measure certain socio-demographic exposures (e.g., level of education, marital status/cohabitation), and variability in the intensity of certain clinical exposures collected (e.g., chemotherapy, radiotherapy, surgery) may have contributed to the high heterogeneity found in our syntheses. In addition, the wide time window used to define RTW outcomes (ranging from 6 months to 2 years) may capture different phases of recovery and work reintegration, potentially reflecting distinct underlying mechanisms. This variability may have further contributed to heterogeneity across studies and should be considered when interpreting the results. The high degree of heterogeneity limits the direct clinical applicability of pooled estimates. While the random-effects approach accounts for statistical variability, it does not fully resolve differences in underlying populations and contexts. Therefore, the results should be interpreted within specific clinical and socio-economic settings. We opted not to adopt a precise threshold value for the income variable due to the heterogeneity of measurement units reported in the various articles included in the analysis. Another factor that may have limited our results is related to the use of only three electronic databases as data sources, which may have obscured some records. Finally, we found a few studies conducted in low-income countries, which affected the findings of our syntheses. Despite these limitations, it is important to highlight several strengths that enhance the consistency of the analyses. In particular, the elevated number of articles reviewed and of exposures considered reinforces the validity of our conclusions, making this study one of the most extensive ever conducted on the topic. Additionally, a key strength is that each meta-analysis was based on a minimum of 7 studies, which not only bolsters the statistical robustness of the results but also minimizes the risk of bias associated with small sample sizes. Moreover, our work adopts a global perspective, encompassing multiple countries and diverse socioeconomic contexts.

## Conclusions

This meta-analysis provides a comprehensive review of existing observational studies on RTW after a cancer diagnosis. Consistent with previous systematic reviews, our findings confirm that the RTW process is influenced by a range of sociodemographic, clinical, and occupational factors. We also found that sex plays a key role, with variations potentially influenced by factors such as treatment differences across cancer types. By exploring the full spectrum of cancer types alongside a wide range of sociodemographic, clinical, psychological, and work-related factors, this analysis offers a novel contribution to the literature. These findings deepen our understanding of the multifaceted nature of RTW and highlight the importance of addressing the process in a personalized way, considering the different aspects of the lives of people who have experienced cancer.

## Data Availability

Publicly available datasets were analyzed in this study. This data can be found here: alberto.catalano@unito.it.
