# Supplementary material for: Risk factors for return to work in workers who experienced cancer: a systematic review with meta-analysis
Source: Oncol Rev. 2026 Jun 16;20:1759182. doi: 10.3389/or.2026.1759182 (PMC13334481; doi:10.3389/or.2026.1759182)
Supplement: Supplementary file 1 [file DataSheet1.docx]

***S1. List of excluded studies.***

| **Excluded studies** | | **Reasons** |
| --- | --- | --- |
| 1 | Abebe E, Giru BW, Boka A. Sleep Quality and Associated Factors Among Adult Cancer Patients on Treatments at Tikur Anbessa Specialized Hospital Oncology Unit, Addis Ababa, Ethiopia, 2021. Cancer Control. 2023;30:10732748231160129. doi:10.1177/10732748231160129 | OUTCOME |
| 2 | Agarwal J, Krishnatry R, Chaturvedi P, et al. Survey of return to work of head and neck cancer survivors: A report from a tertiary cancer center in India. Head Neck. 2017;39(5):893-899. doi:10.1002/hed.24703 | POP |
| 3 | Andreu Y, Picazo C, Murgui S, Soto-Rubio A, García-Conde A, Romero R. Exploring the independent association of employment status to cancer survivors’ health-related quality of life. Health Qual Life Outcomes. 2023;21(1):44. doi:10.1186/s12955-023-02124-y | OUTCOME |
| 4 | Arfi A, Baffert S, Soilly AL, et al. Determinants of return at work of breast cancer patients: results from the OPTISOINS01 French prospective study. BMJ Open. 2018;8(5):e020276. Published 2018 May 18. doi:10.1136/bmjopen-2017-020276 | OUTCOME |
| 5 | Assogba ELF, Kamga AM, Costaz H, et al. What Are Young Women Living Conditions after Breast Cancer? Health-Related Quality of Life, Sexual and Fertility Issues, Professional Reinsertion. Cancers (Basel). 2020;12(6):1564. Published 2020 Jun 12. doi:10.3390/cancers12061564 | OUTCOME |
| 6 | Badreau M, Fadel M, Roquelaure Y, et al. Comparison of Machine Learning Methods in the Study of Cancer Survivors’ Return to Work: An Example of Breast Cancer Survivors with Work-Related Factors in the CONSTANCES Cohort. Journal of Occupational Rehabilitation. 2023;33(4):750-756. doi:10.1007/s10926-023-10112-8 | OTHER |
| 7 | Balakrishnan A, Burdett KB, Kocherginsky M, Jordan N. Racial and ethnic disparities in surgery for kidney cancer: a SEER analysis, 2007-2014. Ethn Health. 2023;28(8):1103-1114. doi:10.1080/13557858.2023.2212145 | OUTCOME |
| 8 | Balhi S, Khiari H, Hsairi M. Factors Associated with Diagnostic Delays among Tunisian Breast Cancer Patients. Asian Pac J Cancer Prev. 2023;24(2):471-477. doi:10.31557/APJCP.2023.24.2.471 | OUTCOME |
| 9 | Barnard EP, AbdElmagied AM, Vaughan LE, et al. Periprocedural outcomes comparing fibroid embolization and focused ultrasound: a randomized controlled trial and comprehensive cohort analysis. Am J Obstet Gynecol. 2017;216(5):500.e1-500.e11. doi:10.1016/j.ajog.2016.12.177 | POP |
| 10 | Behmanesh B, Gessler F, Won SY, et al. Return to work and clinical outcome after surgical treatment and conservative management of patients with intramedullary spinal cord ependymoma. Sci Rep. 2020;10(1):2335. doi:10.1038/s41598-020-59328-1 | OTHER |
| 11 | Berger AK, Chopra S, Desai MM, Aron M, Gill IS. Outpatient Robotic Radical Prostatectomy: Matched-Pair Comparison with Inpatient Surgery. J Endourol. 2016;30 Suppl 1:S52-56. doi:10.1089/end.2016.0135 | OTHER |
| 12 | Berkhout MA, Tamminga SJ, de Boer AGEM, et al. Pilot implementation of MiLES: a web-based intervention targeted at managers with the aim of enhancing the successful return to work of employees with cancer. Acta Oncologica. 2023;62(7):765-773. doi:10.1080/0284186X.2023.2238886 | QUALITATIVE |
| 13 | Bhalla A, Williams JP, Hurst NG, et al. One-third of patients fail to return to work 1 year after surgery for colorectal cancer. Tech Coloproctol. 2014;18(12):1153-1159. doi:10.1007/s10151-014-1232-y | OUTCOME |
| 14 | Bhatt NS, Brazauskas R, Salit RB, et al. Return to Work Among Young Adult Survivors of Allogeneic Hematopoietic Cell Transplantation in the United States. Transplant Cell Ther. 2021;27(8):679.e1-679.e8. doi:10.1016/j.jtct.2021.04.013 | POP |
| 15 | Bier S, Hennenlotter J, Rausch S, et al. Return to Work and Normal Daily Life Activity after Open and Robot-Assisted Radical Prostatectomy--A Single Surgeon Analysis. Urol Int. 2016;96(3):280-286. doi:10.1159/000437335 | OUTCOME |
| 16 | Blinder V, Eberle C, Patil S, Gany FM, Bradley CJ. Women With Breast Cancer Who Work For Accommodating Employers More Likely To Retain Jobs After Treatment. Health Aff (Millwood). 2017;36(2):274-281. doi:10.1377/hlthaff.2016.1196 | OUTCOME |
| 17 | Blinder V, Patil S, Eberle C, Griggs J, Maly RC. Early predictors of not returning to work in low-income breast cancer survivors: a 5-year longitudinal study. Breast Cancer Res Treat. 2013;140(2):407-416. doi:10.1007/s10549-013-2625-8 | AGE |
| 18 | Borgfeldt C, Kalapotharakos G, Asciutto KC, Löfgren M, Högberg T. A population-based registry study evaluating surgery in newly diagnosed uterine cancer. Acta Obstet Gynecol Scand. 2016;95(8):901-911. doi:10.1111/aogs.12918 | OUTCOME |
| 19 | Böttcher HM, Steimann M, Rotsch M, Zurborn KH, Koch U, Bergelt C. Occupational stress and its association with early retirement and subjective need for occupational rehabilitation in cancer patients. Psycho-Oncology. 2013;22(8):1807-1814. doi:10.1002/pon.3224 | OUTCOME |
| 20 | Böttcher HM, Steimann M, Ullrich A, et al. Work-related predictors of not returning to work after inpatient rehabilitation in cancer patients. Acta Oncol. 2013;52(6):1067-1075. doi:10.3109/0284186X.2013.792991 | OUTCOME |
| 21 | Broomfield SJ, Mandavia AK, Nicholson JS, et al. Long-term Quality of Life Following Vestibular Schwannoma Excision Via the Translabyrinthine Approach. Otol Neurotol. 2017;38(8):1165-1173. doi:10.1097/MAO.0000000000001507 | OUTCOME |
| 22 | Bryden P, Paracha N, Thuresson P. Work productivity activity impairment loss of patients with HER2 metastatic breast cancer. Value in health Conference: ISPOR 19th annual european congress Austria Conference start: 20161029 Conference end: 20161102. 2016;19(7):A749. | CONFERENCE |
| 23 | Butenschoen VM, Gloßner T, Hostettler IC, Meyer B, Wostrack M. Quality of life and return to work and sports after spinal ependymoma resection. Sci Rep. 2022;12(1):4926. doi:10.1038/s41598-022-09036-9 | OUTCOME |
| 24 | Carlsen K, Ewertz M, Dalton SO, Badsberg JH, Osler M. Unemployment among breast cancer survivors. Scand J Public Health. 2014;42(3):319-328. doi:10.1177/1403494813520354 | OTHER |
| 25 | Carlsen K, Jensen AJ, Rugulies R, et al. Self-reported work ability in long-term breast cancer survivors. A population-based questionnaire study in Denmark. Acta Oncologica. 2013;52(2):423-429. doi:10.3109/0284186X.2012.744877 | OUTCOME |
| 26 | Chen L, Alexanderson KAE. Trajectories of sickness absence and disability pension in the 2 years before and 3 years after breast cancer diagnosis: A Swedish longitudinal population-based cohort study. Cancer. 2020;126(12):2883-2891. doi:10.1002/cncr.32820 | OUTCOME |
| 27 | Chen L, Glimelius I, Neovius M, et al. Work Loss Duration and Predictors Following Rectal Cancer Treatment among Patients with and without Prediagnostic Work Loss. Cancer Epidemiol Biomarkers Prev. 2016;25(6):987-994. doi:10.1158/1055-9965.EPI-16-0112 | OUTCOME |
| 28 | Chen YJ, Lai YH, Lee YH, Tsai KY, Chen MK, Hsieh MY. Impact of illness perception, mental adjustment, and sociodemographic characteristics on return to work in patients with head and neck cancer. Support Care Cancer. 2021;29(3):1519-1526. doi:10.1007/s00520-020-05640-5 | AGE |
| 29 | Clarke TC, Christ SL, Soler-Vila H, et al. Working with cancer: health and employment among cancer survivors. Annals of Epidemiology. 2015;25(11):832-838. doi:10.1016/j.annepidem.2015.07.011 | OUTCOME |
| 30 | Clin B, Heutte N, Boulanger M, et al. Analysis of medico-social factors for return to work among patients presenting with haematological malignancy (adamantine): results of a “pilot study”. BMC Res Notes. 2020;13(1):313. doi:10.1186/s13104-020-05149-4 | POP |
| 31 | Compaci G, Conte C, Oberic L, Ysebaert L, Laurent G, Despas F. Sustained degradation of quality of life in a subgroup of lymphoma survivors: a two-year prospective survey. BMC Cancer. 2019;19(1):1178. doi:10.1186/s12885-019-6337-2 | OTHER |
| 32 | Cooper AF, Hankins M, Rixon L, Eaton E, Grunfeld EA. Distinct work-related, clinical and psychological factors predict return to work following treatment in four different cancer types. Psycho-Oncology. 2013;22(3):659-667. doi:10.1002/pon.3049 | OTHER |
| 33 | Costa JM, López M, García J, León X, Quer M. Impact of total laryngectomy on return to work. Acta Otorrinolaringol Esp (Engl Ed). 2018;69(2):74-79. doi:10.1016/j.otorri.2017.02.006 | POP |
| 34 | Couwenberg AM, Intven MPW, Gregorowitsch ML, Haaring C, van Grevenstein W, Marieke Verkooijen H. Patient-Reported Work Ability During the First Two Years After Rectal Cancer Diagnosis. Dis Colon Rectum. 2020;63(5):578-587. doi:10.1097/DCR.0000000000001601 | OUTCOME |
| 35 | Creff G, Jegoux F, Bendiane MK, Babin E, Licaj I. Returning to work by thyroid cancer survivors 5 years after diagnosis: the VICAN survey. J Cancer Surviv. 2022;16(4):801-811. doi:10.1007/s11764-021-01074-y | OUTCOME |
| 36 | De Groef A, Van der Gucht E, Devoogdt N, et al. Returning to Work After Breast Cancer Surgery: A Randomised Controlled Trial on the Effect of Pain Neuroscience Education. Journal of Occupational Rehabilitation. 2023;33(4):757-765. doi:10.1007/s10926-023-10103-9 | POP |
| 37 | de Jong M, Tamminga SJ, Frings-Dresen MHW, de Boer AGEM. Quality of Working Life of cancer survivors: associations with health- and work-related variables. Support Care Cancer. 2017;25(5):1475-1484. doi:10.1007/s00520-016-3549-8 | OUTCOME |
| 38 | de Moor JS, Kent EE, Dowling EC, et al. Impact of sociodemographic characteristics on underemployment in a longitudinal, nationally representative study of cancer survivors: Evidence for the importance of gender and marital status. Journal of Psychosocial Oncology. 2018;36(3):287-303. doi:10.1080/07347332.2018.1440274 | POP |
| 39 | de Souza Cunha N, Zomkowski K, Fernandes BL, Sacomori C, de Azevedo Guimarães AC, Sperandio FF. Physical symptoms and components of labor tasks associated with upper limb disability among working breast cancer survivors. Breast Cancer. 2020;27(1):140-146. doi:10.1007/s12282-019-01004-y | OTHER |
| 40 | Di Meglio A, Menvielle G, Dumas A, et al. Impact of overweight, obesity, and post-treatment weight changes on occupational reintegration of breast cancer (BC) survivors. Journal of clinical oncology. 2019;37. doi:10.1200/JCO.2019.37.15_suppl.11562 | CONFERENCE |
| 41 | Dorland HF, Abma FI, Roelen CAM, et al. Work-specific cognitive symptoms and the role of work characteristics, fatigue, and depressive symptoms in cancer patients during 18 months post return to work. Psycho-Oncology. 2018;27(9):2229-2236. doi:10.1002/pon.4800 | OUTCOME |
| 42 | Dorland HF, Abma FI, Van Zon SKR, et al. Fatigue and depressive symptoms improve but remain negatively related to work functioning over 18 months after return to work in cancer patients. Journal of Cancer Survivorship. 2018;12(3):371-378. doi:10.1007/s11764-018-0676-x | OUTCOME |
| 43 | Duijts SFA, van der Beek AJ, Bleiker EMA, Smith L, Wardle J. Cancer and heart attack survivors’ expectations of employment status: results from the English Longitudinal Study of Ageing. BMC Public Health. 2017;17:1-10. doi:10.1186/s12889-017-4659-z | OTHER |
| 44 | Eikeland SA, Smeland KB, Simensen VC, et al. Chronic fatigue in long-term survivors of Hodgkin’s lymphoma after contemporary risk-adapted treatment. Acta Oncol. 2023;62(1):80-88. doi:10.1080/0284186X.2023.2168215 | OUTCOME |
| 45 | Ekenga CC, Kim B, Kwon E, Park S. Multimorbidity and Employment Outcomes Among Middle-Aged US Cancer Survivors. Journal of Occupational & Environmental Medicine. 2022;64(6):476-481. doi:10.1097/JOM.0000000000002473 | OTHER |
| 46 | Ekenga CC, Kwon E, Kim B, Park S. Long-Term Employment Outcomes among Female Cancer Survivors. Int J Environ Res Public Health. 2020;17(8). doi:10.3390/ijerph17082751 | OTHER |
| 47 | Ekenga CC, Pérez M, Margenthaler JA, Jeffe DB. Early-stage breast cancer and employment participation after 2 years of follow-up: A comparison with age-matched controls. Cancer (0008543X). 2018;124(9):2026-2035. doi:10.1002/cncr.31270 | OTHER |
| 48 | Endo M, Haruyama Y, Muto G, et al. Work Sustainability Among Male Cancer Survivors After Returning to Work. J Epidemiol. 2018;28(2):88-93. doi:10.2188/jea.JE20160152 | OUTCOME |
| 49 | Fatobene G, Storer BE, Salit RB, et al. Disability related to chronic graft -versus-host disease after alternative donor hematopoietic cell transplantation. Haematologica. 2019;104(4):835-843. doi:10.3324/haematol.2018.202754 | OUTCOME |
| 50 | Fauser D, Wienert J, Zomorodbakhsch B, et al. Work-Related Medical Rehabilitation in Cancer: a Cluster-Randomized Multicenter Study. Deutsches Arzteblatt international. 2019;116(35‐36):592‐599. doi:10.3238/arztebl.2019.0592 | OTHER |
| 51 | Ferrier C, Thebaut C, Levy P, et al. Absenteeism and indirect costs during the year following the diagnosis of an operable breast cancer: A prospective multicentric cohort study. J Gynecol Obstet Hum Reprod. 2021;50(6):101871. doi:10.1016/j.jogoh.2020.101871 | OTHER |
| 52 | Fitch MI, Lockwood G, Nicoll I. Physical, emotional, and practical concerns, help-seeking and unmet needs of rural and urban dwelling adult cancer survivors. European Journal of Oncology Nursing. 2021;53:N.PAG-N.PAG. doi:10.1016/j.ejon.2021.101976 | OUTCOME |
| 53 | Ganem G, Antoine E‐ C, Touboul C, et al. Maintaining professional activity during breast cancer treatment. European Journal of Cancer Care. 2016;25(3):458-465. doi:10.1111/ecc.12460 | OUTCOME |
| 54 | Gardiner R, Yaxley J, Coughlin G, et al. A randomized controlled trial of robotic versus open radical prostatectomy: early outcomes. Asia-Pacific journal of clinical oncology. 2016;12:29‐. doi:10.1111/ajco.12557 | CONFERENCE |
| 55 | Geeraerts M, Silva Corten LC, van Det M, et al. Insights in work rehabilitation after minimally invasive esophagectomy. Surgical Endoscopy. 2019;33(10):3457-3463. doi:10.1007/s00464-018-06626-5 | OUTCOME |
| 56 | Ghasempour M, Rahmani A, Davoodi A, Sheikhalipour Z, Ziaeei JE, Abri F. Return to work and its relation to financial distress among Iranian cancer survivors. Asian Pac J Cancer Prev. 2015;16(6):2257-2261. doi:10.7314/apjcp.2015.16.6.2257 | OTHER |
| 57 | Ghasempour M, Shabanloei R, Rahmani A, Jafarabadi MA, Abri F, Khajehgoodari M. The Relation of Readiness for Return to Work and Return to Work Among Iranian Cancer Survivors. Journal of Cancer Education. 2020;35(6):1237-1242. doi:10.1007/s13187-019-01588-1 | OUTCOME |
| 58 | Giuliani M, Papadakos J, Broadhurst M, et al. The prevalence and determinants of return to work in head and neck cancer survivors. Support Care Cancer. 2019;27(2):539-546. doi:10.1007/s00520-018-4343-6 | OUTCOME |
| 59 | Grabowski DC, Kansal AR, Goldman DP, Lakdawalla DN. Assessment of Medical and Public Assistance Expenditures and Employment Among US Adults With Cancer Diagnoses. JAMA Netw Open. 2023;6(5):e2315823. doi:10.1001/jamanetworkopen.2023.15823 | OUTCOME |
| 60 | Gragnano A, Miglioretti M, Magon G, Pravettoni G. Work with cancer or stop working after diagnosis? Variables affecting the decision. Work. 2021;70(1):177-185. doi:10.3233/WOR-213563 | OUTCOME |
| 61 | Granstam B, Rosenblad A, Lindemalm C, et al. A randomized controlled trial of support group intervention after breast cancer treatment: results on sick leave, health care utilization and health economy. Cancer research. 2012;72(24). doi:10.1158/0008-5472.SABCS12-P2-12-09 | OTHER |
| 62 | Grinshpun A, Rottenberg Y. Unemployment following breast cancer diagnosis: A population-based study. Breast. 2019;44:24-28. doi:10.1016/j.breast.2018.12.013 | OUTCOME |
| 63 | Grunfeld EA, Schumacher L, Armaou M, et al. Feasibility randomised controlled trial of a guided workbook intervention to support work-related goals among cancer survivors in the UK. BMJ Open. 2019;9(1):e022746. doi:10.1136/bmjopen-2018-022746 | AGE |
| 64 | Guadamuz JS, Wang X, Ryals CA, et al. Socioeconomic status and inequities in treatment initiation and survival among patients with cancer, 2011-2022. JNCI Cancer Spectr. 2023;7(5). doi:10.1093/jncics/pkad058 | OUTCOME |
| 65 | Hartung TJ, Sautier LP, Scherwath A, et al. Return to Work in Patients with Hematological Cancers 1 Year after Treatment: A Prospective Longitudinal Study. Oncol Res Treat. 2018;41(11):697-701. doi:10.1159/000491589 | OTHER |
| 66 | Hass H, Muthny F, Stepien J, et al. Effects of a Phone-Based Follow-Up Care After Inpatient Rehabilitation for Breast Cancer Patients - A Randomized Controlled Trial. Die Rehabilitation. 2017;56(3):189‐197. doi:10.1055/s-0042-121384 | LANGUAGE |
| 67 | Hastert TA, Kirchhoff AC, Banegas MP, et al. Work changes and individual, cancer-related, and work-related predictors of decreased work participation among African American cancer survivors. Cancer Med. 2020;9(23):9168-9177. doi:10.1002/cam4.3512 | AGE |
| 68 | Hauglann BK, Saltytė Benth J, Fosså SD, Tveit KM, Dahl AA. A controlled cohort study of sickness absence and disability pension in colorectal cancer survivors. Acta Oncol. 2014;53(6):735-743. doi:10.3109/0284186X.2013.844354 | OUTCOME |
| 69 | Heiman J, Pavia J, Bock D, Haglind E, Olofsson Bagge R. Sick leave and predictive factors for sick leave at 12 months after breast cancer surgery in the randomized controlled physical activity trial (PhysSURG-B). Surgeon. 2023;21(5):e229-e237. doi:10.1016/j.surge.2022.12.001 | AGE |
| 70 | Hernæs KH, Smeland KB, Fagerli UM, Kiserud CE. Post-treatment work patterns amongst survivors of lymphoma treated with high-dose chemotherapy with autologous stem-cell transplantation. BMC Cancer. 2021;21(1):1-11. doi:10.1186/s12885-021-07836-2 | OUTCOME |
| 71 | Horsboel TA, Nielsen CV, Nielsen B, Andersen NT, De Thurah A. Wage-subsidised employment as a result of permanently reduced work capacity in a nationwide cohort of patients diagnosed with haematological malignancies. Acta Oncol. 2015;54(5):743-749. doi:10.3109/0284186X.2014.999871 | POP |
| 72 | Hu L, Zhao JS, Xing C, et al. Comparison of Focused Ultrasound Surgery and Hysteroscopic Resection for Treatment of Submucosal Uterine Fibroids (FIGO Type 2). Ultrasound Med Biol. 2020;46(7):1677-1685. doi:10.1016/j.ultrasmedbio.2020.02.018 | OTHER |
| 73 | Huff KO, Aref‐Adib M, Magama Z, et al. Returning to work after laparoscopic myomectomy: a prospective observational study. Acta Obstetricia et Gynecologica Scandinavica. 2018;97(1):68-73. doi:10.1111/aogs.13246 | OTHER |
| 74 | Ibrahim M, Muanza T, Smirnow N, et al. Time course of upper limb function and return-to-work post-radiotherapy in young adults with breast cancer: a pilot randomized control trial on effects of targeted exercise program. Journal of Cancer Survivorship. 2017;11(6):791-799. doi:10.1007/s11764-017-0617-0 | OTHER |
| 75 | Isaksson J, Wilms T, Laurell G, Fransson P, Ehrsson YT. Meaning of work and the process of returning after head and neck cancer. Support Care Cancer. 2016;24(1):205-213. doi:10.1007/s00520-015-2769-7 | AGE |
| 76 | Jagsi R, Abrahamse PH, Lee KL, et al. Treatment decisions and employment of breast cancer patients: Results of a population-based survey. Cancer (0008543X). 2017;123(24):4791-4799. doi:10.1002/cncr.30959 | AGE |
| 77 | Jagsi R, Hawley ST, Abrahamse P, et al. Impact of adjuvant chemotherapy on long-term employment of survivors of early-stage breast cancer. Cancer (0008543X). 2014;120(12):1854-1862. doi:10.1002/cncr.28607 | AGE |
| 78 | Jensen LS, Overgaard C, Garne JP, Bøggild H, Fonager K. The impact of prior psychiatric medical treatment on return to work after a diagnosis of breast cancer: A registry based study. Scandinavian Journal of Public Health. 2019;47(5):519-527. doi:10.1177/1403494817722291 | OTHER |
| 79 | Jolly PE, Junkins A, Aung M. Sociodemographic characteristics, attitudes, and knowledge associated with previous screening for cervical cancer among women in western Jamaica. Infectious Agents & Cancer. 2023;18(1):1-11. doi:10.1186/s13027-023-00537-4 | OUTCOME |
| 80 | Jong M, Boers I, Schouten van der Velden A, et al. A Randomized Study of Yoga for Fatigue and Quality of Life in Women with Breast Cancer Undergoing (Neo) Adjuvant Chemotherapy. Journal of alternative and complementary medicine (New York, NY). 2018;24(9‐10):942‐953. doi:10.1089/acm.2018.0191 | OTHER |
| 81 | Juul SJ, Rossetti S, Kicinski M, et al. Work and education interruption in long-term Hodgkin lymphoma survivors: an analysis among patients from nine EORTC-LYSA trials. Acta Oncologica. 2023;62(7):744-752. doi:10.1080/0284186X.2023.2195561 | AGE |
| 82 | Kang D, Bae K, Ahn Y, et al. Effectiveness of Self-Assessment, TAilored Information, and Lifestyle Management for Cancer Patients’ Returning to Work (START): a Multi-center, Randomized Controlled Trial. Cancer research and treatment. Published online 2022. doi:10.4143/crt.2022.939 | AGE |
| 83 | Kang D, Bae K, Ahn Y, et al. Effectiveness of Self-Assessment, TAilored Information, and Lifestyle Management for Cancer Patients’ Returning to Work (START): a Multi-center, Randomized Controlled Trial. Cancer research and treatment. Published online 2022. doi:10.4143/crt.2022.939 | AGE |
| 84 | Kelsall JE, McCulley SJ, Brock L, Akerlund MTE, Macmillan RD. Comparing oncoplastic breast conserving surgery with mastectomy and immediate breast reconstruction: Case-matched patient reported outcomes. J Plast Reconstr Aesthet Surg. 2017;70(10):1377-1385. doi:10.1016/j.bjps.2017.05.009 | OTHER |
| 85 | Kenborg L, Frederiksen LE, Galanakis M, et al. Employment, occupation, and income in adults with neurofibromatosis 1 in Denmark: a population- and register-based cohort study. Orphanet J Rare Dis. 2023;18(1):346. doi:10.1186/s13023-023-02965-2 | OTHER |
| 86 | Ketterl TG, Syrjala KL, Casillas J, et al. Lasting effects of cancer and its treatment on employment and finances in adolescent and young adult cancer survivors. Cancer. 2019;125(11):1908-1917. doi:10.1002/cncr.31985 | POP |
| 87 | Kiasuwa Mbengi RL, Nicolaie AM, Goetghebeur E, et al. Assessing factors associated with long-term work disability after cancer in Belgium: a population-based cohort study using competing risks analysis with a 7-year follow-up. BMJ Open. 2018;8(2):e014094. doi:10.1136/bmjopen-2016-014094 | OUTCOME |
| 88 | Kim YA, Yun YH, Chang YJ, et al. Employment status and work-related difficulties in lung cancer survivors compared with the general population. Ann Surg. 2014;259(3):569-575. doi:10.1097/SLA.0b013e318291db9d | POP |
| 89 | Kircher S, Duan F, An N, et al. Patient-Reported Financial Burden of Treatment for Colon or Rectal Cancer. JAMA Network Open. 2024;7(1):e2350844-e2350844. doi:10.1001/jamanetworkopen.2023.50844 | OUTCOME |
| 90 | Kisook Kim, Booyoung Oh. Factors Associated with Stress among Employed Korean Cancer Survivors: A Cross-sectional Descriptive Study Based on National Data. Korean Journal of Adult Nursing. 2023;35(3):254-263. doi:10.7475/kjan.2023.35.3.254 | OUTCOME |
| 91 | Kjær T, Bøje CR, Olsen MH, et al. Affiliation to the work market after curative treatment of head-and-neck cancer: A population-based study from the DAHANCA database...Danish Head and Neck Cancer Group. Acta Oncologica. 2013;52(2):430-439. doi:10.3109/0284186X.2012.746469 | POP |
| 92 | Koch R, Wittekindt C, Altendorf-Hofmann A, Singer S, Guntinas-Lichius O. Employment pathways and work-related issues in head and neck cancer survivors. Head Neck. 2015;37(4):585-593. doi:10.1002/hed.23640 | POP |
| 93 | Kolodziejczyk C, Heinesen E. Labour market participation after breast cancer for employees from the private and public sectors: Educational and sector gradients in the effect of cancer. Econ Hum Biol. 2016;21:33-55. doi:10.1016/j.ehb.2015.12.002 | OUTCOME |
| 94 | Krimphove MJ, Reese SW, Chen X, et al. Recovery from minimally invasive vs. open surgery in kidney cancer patients: Opioid use and workplace absenteeism. Investig Clin Urol. 2021;62(1):56-64. doi:10.4111/icu.20200194 | OTHER |
| 95 | Kvillemo PK, Chen L, Bottai M, et al. Sickness absence and disability pension among women with breast cancer: a population-based cohort study from Sweden. BMC Public Health. 2021;21(1):1-12. doi:10.1186/s12889-021-10703-1 | OUTCOME |
| 96 | Langer D, Tendler S, Bar-Haim Erez A. A broad perspective on breast cancer: Participation, quality of life and return to work throughout the recovery process. Work. 2023;75(1):325-337. doi:10.3233/WOR-220085 | OUTCOME |
| 97 | Laughlin-Tommaso SK, Lu D, Thomas L, et al. Short-term quality of life after myomectomy for uterine fibroids from the COMPARE-UF Fibroid Registry. Am J Obstet Gynecol. 2020;222(4):345.e1-345.e22. doi:10.1016/j.ajog.2019.09.052 | OTHER |
| 98 | Law C, Brewer K, Brown C, et al. Return to work following laparoscopic-assisted resection or open resection for rectal cancer: findings from AlaCaRT-Australasian Laparoscopic Cancer of the Rectum Trial. Cancer medicine. 2021;10(2):552‐562. doi:10.1002/cam4.3623 | AGE |
| 99 | Lawlor ER, Donnelly M. Use of an employment advisory service by cancer survivors. J Psychosoc Oncol. 2015;33(3):219-231. doi:10.1080/07347332.2015.1019658 | QUALITATIVE |
| 100 | Lee LH, Yang CI, Chen MK, Hsieh MY, Chen YJ. Exploring return to work for patients with head and neck cancer. Work. 2024;77(1):231-241. doi:10.3233/WOR-220707 | OTHER |
| 101 | Lee MK, Kang HS, Lee KS, Lee ES. Three-Year Prospective Cohort Study of Factors Associated with Return to Work After Breast Cancer Diagnosis. J Occup Rehabil. 2017;27(4):547-558. doi:10.1007/s10926-016-9685-7 | OTHER |
| 102 | Lee MK, Yun YH. Working situation of cancer survivors versus the general population. J Cancer Surviv. 2015;9(2):349-360. doi:10.1007/s11764-014-0418-7 | POP |
| 103 | Lee S, Logan B, Westervelt P, et al. Comparison of Patient-Reported Outcomes in 5-Year Survivors Who Received Bone Marrow vs Peripheral Blood Unrelated Donor Transplantation: long-term Follow-up of a Randomized Clinical Trial. JAMA oncology. 2016;2(12):1583‐1589. doi:10.1001/jamaoncol.2016.2520 | OTHER |
| 104 | Lee YH, Chang KH, Escorpizo R, et al. Accuracy of the World Health Organization Disability Assessment Schedule 2.0 (WHODAS 2.0) score as an objective assessment tool for predicting return-to-work status after head and neck cancer in male survivors. Support Care Cancer. 2019;27(2):433-441. doi:10.1007/s00520-018-4322-y | OTHER |
| 105 | Leeper HE, Vera E, Christ A, et al. Association of Employment Status With Symptom Burden and Health-Related Quality of Life in People Living With Primary CNS Tumors. Neurology. 2023;100(16):e1723-e1736. doi:10.1212/WNL.0000000000207082 | POP |
| 106 | Leskelä RL, Haavisto I, Pennanen P, Lahelma M, Mattson J, Poikonen-Saksela P. Predictive factors for prolonged sick leave in breast cancer patients treated with adjuvant therapies: a retrospective registry study. Acta Oncologica. 2023;62(10):1331-1337. doi:10.1080/0284186X.2023.2254483 | OUTCOME |
| 107 | Leuteritz K, Friedrich M, Sender A, et al. Return to Work and Employment Situation of Young Adult Cancer Survivors: Results from the Adolescent and Young Adult-Leipzig Study. Journal of Adolescent & Young Adult Oncology. 2021;10(2):226-233. doi:10.1089/jayao.2020.0055 | POP |
| 108 | Lewis J, Mackenzie L, Black D. Workforce participation of Australian women with breast cancer. Psycho-Oncology. 2020;29(7):1156-1164. doi:10.1002/pon.5392 | POP |
| 109 | Lieb M, Wünsch A, Schieber K, et al. Return to work after cancer: Improved mental health in working cancer survivors. Psychooncology. 2022;31(6):893-901. doi:10.1002/pon.5877 | OTHER |
| 110 | Lim ZW, Wang CC, Wu WT, Chen WL. Return to Work in Survivors With Occupational Cancers. Journal of Occupational & Environmental Medicine. 2022;64(2):158-165. doi:10.1097/JOM.0000000000002381 | AGE |
| 111 | Luis I, aO’Neill A, Sepucha K, et al. Symptom burden and employment status in breast cancer (BC) survivors. Journal of clinical oncology. 2018;36(15). doi:10.1200/JCO.2018.36.15-suppl.10073 | CONFERENCE |
| 112 | Luker K, Campbell M, Amir Z, Davies L. A UK survey of the impact of cancer on employment. Occup Med (Lond). 2013;63(7):494-500. doi:10.1093/occmed/kqt104 | POP |
| 113 | Lynch B, Mihala G, Beesley V, et al. Associations of health behaviours with return to work outcomes after colorectal cancer. Supportive Care in Cancer. 2016;24(2):865-870. doi:10.1007/s00520-015-2855-x | OTHER |
| 114 | Macias D, Hand BN, Maurer S, et al. Factors Associated With Risk of Body Image-Related Distress in Patients With Head and Neck Cancer. JAMA Otolaryngology-Head & Neck Surgery. 2021;147(12):1019-1026. doi:10.1001/jamaoto.2021.1378 | OUTCOME |
| 115 | Magnavita N, Di Prinzio RR, Meraglia I, et al. Supporting Return to Work after Breast Cancer: A Mixed Method Study. Healthcare (2227-9032). 2023;11(16):2343. doi:10.3390/healthcare11162343 | OUTCOME |
| 116 | Mamguem Kamga A, Dumas A, Joly F, et al. Socio‐economic and occupational outcomes of long‐term survivors of gynaecological cancer: A French population‐based study. European Journal of Cancer Care. 2020;29(4):1-9. doi:10.1111/ecc.13235 | POP |
| 117 | Månsson T, Lynøe N, Alexanderson K, Hinas E, Helgesson G, Friberg E. Respectful encounters from healthcare professionals and return to work among 9032 long-term sick-listed due to cancer or due to other diagnoses: results from a Swedish population-based survey. Supportive Care in Cancer. 2019;27(9):3555-3561. doi:10.1007/s00520-019-4652-4 | OUTCOME |
| 118 | Maraldo M, Kicinski M, Van Der Kaaij M, et al. Employment situation among long-term Hodgkin lymphoma survivors in Europe: an analysis of patients from nine consecutive eortclysa trials. Hemasphere. 2018;2(3):15‐. doi:10.1097/01.HS9.0000547882.25717.7c | OUTCOME |
| 119 | Marino P, Luis Sagaon T, Laetitia M, et al. Sex differences in the return-to-work process of cancer survivors 2 years after diagnosis: results from a large French population-based sample. Journal of Clinical Oncology. 2013;31(10):1277-1284. doi:10.1200/JCO.2011.38.5401 | POP |
| 120 | Markovic C, Mackenzie L, Lewis J, Singh M. Working with cancer: A pilot study of work participation among cancer survivors in Western Sydney. Aust Occup Ther J. 2020;67(6):592-604. doi:10.1111/1440-1630.12685 | POP |
| 121 | Mijwel S, Jervaeus A, Bolam K, et al. High-intensity exercise during chemotherapy induces beneficial effects 12 months into breast cancer survivorship. Journal of cancer survivorship. 2019;13(2):244‐256. doi:10.1007/s11764-019-00747-z | AGE |
| 122 | Mitsui K, Endo M, Imai Y, et al. Predictors of resignation and sick leave after cancer diagnosis among Japanese breast cancer survivors: a cross-sectional study. BMC Public Health. 2021;21(1):1-9. doi:10.1186/s12889-021-10168-2 | AGE |
| 123 | Monteiro, I.; Morais, S.; Costa, A. R.; Lopes-Conceição, L.; Araújo, N.; Fontes, F.; Dias, T.; Pereira, S.; Lunet, N. Changes in Employment Status up to 5 Years after Breast Cancer Diagnosis: A Prospective Cohort Study. Breast 2019, 48, 38–44. https://doi.org/10.1016/j.breast.2019.07.007 | OTHER |
| 124 | Müller G, Butea-Bocu MC, Beyer B, et al. Prospective evaluation of return to work, health-related quality of life and psychosocial distress after radical cystectomy: 1-year follow-up in 230 employed German bladder cancer patients. World J Urol. 2023;41(10):2707-2713. doi:10.1007/s00345-023-04570-1 | POP |
| 125 | Murdaugh DL, Bosworth A, Patel SK, et al. Self-endorsed cognitive problems versus objectively assessed cognitive impairment in blood or bone marrow transplantation recipients: A longitudinal study. Cancer. 2020;126(10):2174-2182. doi:10.1002/cncr.32773 | AGE |
| 126 | Murray K, Lam KBH, McLoughlin D, Sadhra SS. Factors in Removing Job Restrictions for Cancer Survivors in the United Kingdom Royal Air Force. J Occup Rehabil. 2019;29(1):25-30. doi:10.1007/s10926-018-9758-x | OUTCOME |
| 127 | Musti MA, Collina N, Stivanello E, et al. Perceived work ability at return to work in women treated for breast cancer: a questionnaire-based study. Med Lav. 2018;109(6):407-419. doi:10.23749/mdl.v110i6.7241 | AGE |
| 128 | Muto J, Dezamis E, Rigaux-Viode O, et al. Functional-Based Resection Does Not Worsen Quality of Life in Patients with a Diffuse Low-Grade Glioma Involving Eloquent Brain Regions: A Prospective Cohort Study. World Neurosurg. 2018;113:e200-e212. doi:10.1016/j.wneu.2018.01.213 | OUTCOME |
| 129 | Nakamura K, Masuyama H, Ida N, et al. Radical Hysterectomy Plus Concurrent Chemoradiation/Radiation Therapy Is Negatively Associated With Return to Work in Patients With Cervical Cancer. Int J Gynecol Cancer. 2017;27(1):117-122. doi:10.1097/IGC.0000000000000840 | OTHER |
| 130 | Nakamura K, Masuyama H, Nishida T, et al. Return to work after cancer treatment of gynecologic cancer in Japan. BMC Cancer. 2016;16:558. doi:10.1186/s12885-016-2627-0 | OTHER |
| 131 | Ng S, Herbet G, Moritz-Gasser S, Duffau H. Return to Work Following Surgery for Incidental Diffuse Low-Grade Glioma: A Prospective Series With 74 Patients. Neurosurgery. 2020;87(4):720-729. doi:10.1093/neuros/nyz513 | OUTCOME |
| 132 | Nikita, Rani R, Kumar R. Body image distress among cancer patients: needs for psychosocial intervention development. Support Care Cancer. 2022;30(7):6035-6043. doi:10.1007/s00520-022-07049-8 | OUTCOME |
| 133 | Nilsson MI, Petersson LM, Wennman-Larsen A, Olsson M, Vaez M, Alexanderson K. Adjustment and social support at work early after breast cancer surgery and its associations with sickness absence. Psycho-Oncology. 2013;22(12):2755-2762. doi:10.1002/pon.3341 | OUTCOME |
| 134 | Nilsson R, Dahl AA, Bernklev T, Kersten H, Haug ES. Work status and work ability after radical prostatectomy or active surveillance for prostate cancer. Scand J Urol. 2020;54(3):194-200. doi:10.1080/21681805.2020.1750473 | AGE |
| 135 | Nitecki R, Fu S, Lefkowits C, et al. Employment disruption following the diagnosis of endometrial cancer. Gynecol Oncol. 2021;160(1):199-205. doi:10.1016/j.ygyno.2020.10.041 | OUTCOME |
| 136 | Noeres D, Park-Simon TW, Grabow J, et al. Return to work after treatment for primary breast cancer over a 6-year period: results from a prospective study comparing patients with the general population. Supportive Care in Cancer. 2013;21(7):1901-1909. doi:10.1007/s00520-013-1739-1 | AGE |
| 137 | Nord C, Olofsson SE, Glimelius I, et al. Sick leave and disability pension among Swedish testicular cancer survivors according to clinical stage and treatment. Acta Oncol. 2015;54(10):1770-1780. doi:10.3109/0284186X.2015.1020967 | OUTCOME |
| 138 | Okeke B, Hillmon C, Jones J, et al. The relationship of social determinants and distress in newly diagnosed cancer patients. Sci Rep. 2023;13(1):2153. doi:10.1038/s41598-023-29375-5 | OUTCOME |
| 139 | Özer Güçlüel Y, Can G. Evaluation of job stress and quality of life in individuals returning to work after cancer treatment. European Journal of Oncology Nursing. 2023;66:N.PAG-N.PAG. doi:10.1016/j.ejon.2023.102381 | OUTCOME |
| 140 | Paltrinieri S, Vicentini M, Mancuso P, et al. Return to work of Italian cancer survivors: A focus on prognostic work-related factors. Work. 2022;71(3):681-691. doi:10.3233/WOR-210008 | OTHER |
| 141 | Parsons HM, Harlan LC, Lynch CF, et al. Impact of cancer on work and education among adolescent and young adult cancer survivors. Journal of Clinical Oncology. 2012;30(19):2393-2400. doi:10.1200/JCO.2011.39.6333 | AGE |
| 142 | Pauwels EE, Charlier C, De Bourdeaudhuij I, Lechner L, Van Hoof E. Care needs after primary breast cancer treatment. Survivors’ associated sociodemographic and medical characteristics. Psycho-Oncology. 2013;22(1):125-132. doi:10.1002/pon.2069 | OTHER |
| 143 | Peipins LA, Dasari S, Rodriguez JL, White MC, Hodgson ME, Sandler DP. Employment After Breast Cancer Diagnosis and Treatment Among Women in the Sister and the Two Sister Studies. Journal of Occupational Rehabilitation. 2021;31(3):543-551. doi:10.1007/s10926-020-09951-6 | AGE |
| 144 | Petersson L, Vaez M, Nilsson MI, et al. Sickness absence following breast cancer surgery: a two‐year follow‐up cohort study. Scandinavian Journal of Caring Sciences. 2018;32(2):715-724. doi:10.1111/scs.12502 | OTHER |
| 145 | Petersson LM, Nilsson MI, Alexanderson K, Olsson M, Wennman-Larsen A. How do women value work shortly after breast cancer surgery and are their valuations associated with being on sick leave? J Occup Rehabil. 2013;23(3):391-399. doi:10.1007/s10926-012-9402-0 | OUTCOME |
| 146 | Picazo C, Gil-Juliá B, Soto-Rubio A, et al. Returning to work in cancer survivors: a multi-center cross-sectional study in Spain. Psicooncologia. 2023;20(1):139-153. doi:10.5209/psic.87574 | OTHER |
| 147 | Pinto E, Cavallin F, Alfieri R, et al. Impact of esophagectomy for cancer on patients’ occupational status. Eur J Surg Oncol. 2016;42(1):103-109. doi:10.1016/j.ejso.2015.09.021 | AGE |
| 148 | Plym A, Chiesa F, Voss M, et al. Work Disability After Robot-assisted or Open Radical Prostatectomy: A Nationwide, Population-based Study. Eur Urol. 2016;70(1):64-71. doi:10.1016/j.eururo.2015.12.049 | OUTCOME |
| 149 | Porro B, Michel A, Baussard L, et al. Quality of life, fatigue and changes therein as predictors of return to work during breast cancer treatment. Scandinavian Journal of Caring Sciences. 2019;33(2):467-477. doi:10.1111/scs.12646 | OTHER |
| 150 | Rai KS, Mann U, Harasemiw O, et al. A prospective evaluation of patient perspectives and financial considerations during prostate cancer treatment decision-making. Canadian Urological Association Journal. 2023;17(9):E244-E251. doi:10.5489/cuaj.8228 | OUTCOME |
| 151 | Ratzon NZ, Uziely B, de Boer AGEM, Rottenberg Y. Unemployment Risk and Decreased Income Two and Four Years After Thyroid Cancer Diagnosis: A Population-Based Study. Thyroid. 2016;26(9):1251-1258. doi:10.1089/thy.2015.0608 | POP |
| 152 | Regenbogen SE, Veenstra CM, Hawley ST, et al. The personal financial burden of complications after colorectal cancer surgery. Cancer. 2014;120(19):3074-3081. doi:10.1002/cncr.28812 | OTHER |
| 153 | Rolland AL, Porro B, Kab S, Ribet C, Roquelaure Y, Bertin M. Impact of breast cancer care pathways and related symptoms on the return-to-work process: results from a population-based French cohort study (CONSTANCES). Breast Cancer Res. 2023;25(1):30. doi:10.1186/s13058-023-01623-6 | AGE |
| 154 | Rollin L, De Blasi G, Boucher L, Bouteyre E, Gehanno JF. Return-to-work support in cancer patients: Which methodology? Bull Cancer. 2020;107(2):200-208. doi:10.1016/j.bulcan.2019.10.006 | POP |
| 155 | Rosbjerg R, Zachariae R, Hansen DG, et al. Physical activity, return to work self-efficacy, and work status among employees undergoing chemotherapy for cancer - a prospective study with 12 months  follow-up. BMC Cancer. 2021;21(1):169. doi:10.1186/s12885-021-07824-6 | OTHER |
| 156 | Rosenberg SM, Vaz-Luis I, Gong J, et al. Employment trends in young women following a breast cancer diagnosis. Breast Cancer Res Treat. 2019;177(1):207-214. doi:10.1007/s10549-019-05293-x | POP |
| 157 | Rottenberg Y, de Boer AGEM. Risk for unemployment at 10 years following cancer diagnosis among very long-term survivors: a population based study. Journal of Cancer Survivorship. 2020;14(2):151-157. doi:10.1007/s11764-020-00858-y | POP |
| 158 | Rottenberg Y, Jacobs J, Ratzon N, et al. Unemployment risk 2 years and 4 years following gastric cancer diagnosis: a population-based study. Journal of Cancer Survivorship. 2017;11(1):119-125. doi:10.1007/s11764-016-0568-x | POP |
| 159 | Rottenberg Y, Ratzon NZ, Cohen M, Hubert A, Uziely B, de Boer AGEM. Unemployment risk at 2 and 4 years following colorectal cancer diagnosis: a population based study. European Journal of Cancer. 2016;69:70-76. doi:10.1016/j.ejca.2016.09.025 | POP |
| 160 | Rottenberg Y, Ratzon NZ, Jacobs JM, Cohen M, Peretz T, de Boer AGEM. Unemployment risk and income change after testicular cancer diagnosis: A population-based study. Urologic Oncology. 2016;34(1):5.e27-5.e33. doi:10.1016/j.urolonc.2015.08.016 | POP |
| 161 | Rudolfsen JH, Hjortsø MD, Pedersen MH, Pilgaard T, Pøhl M. Impact of new cancer medicine – real-world evidence from Danish register study of lung cancer patients. Acta Oncologica. 2023;62(3):253-260. doi:10.1080/0284186X.2023.2185104 | OUTCOME |
| 162 | Samadi O, Breunis H, Sandoval J, Akilan K, Timilshina N, Alibhai SMH. Return to work and work-related disability among AML survivors. Ann Hematol. 2017;96(10):1625-1633. doi:10.1007/s00277-017-3097-4 | AGE |
| 163 | Schucht P, Ghareeb F, Duffau H. Surgery for low-grade glioma infiltrating the central cerebral region: location as a predictive factor for neurological deficit, epileptological outcome, and quality of life. J Neurosurg. 2013;119(2):318-323. doi:10.3171/2013.5.JNS122235 | AGE |
| 164 | Senft C, Behrens M, Lortz I, et al. The ability to return to work: a patient-centered outcome parameter following glioma surgery. J Neurooncol. 2020;149(3):403-411. doi:10.1007/s11060-020-03609-2 | POP |
| 165 | Shabangu N, Thebe T, Casey M, Wesselmann U, Parker R. Chronic pain in female breast cancer survivors - prevalence, characteristics and contributing factors: a cross-sectional pilot study. BMC Womens Health. 2023;23(1):613. doi:10.1186/s12905-023-02766-6 | OUTCOME |
| 166 | Sheppard DM, O’Connor M, Jefford M, et al. “Beyond Cancer” Rehabilitation Program to Support Breast Cancer Survivors to Return to Health, Wellness and Work: Feasibility Study Outcomes. Curr Oncol. 2023;30(2):2249-2270. doi:10.3390/curroncol30020174 | DESIGN |
| 167 | Silvaggi F, Mariniello A, Leonardi M, et al. Psychosocial factors associated with workability after surgery in cancer survivors: An explorative study. Journal of Health Psychology. 2023;28(11):999-1010. doi:10.1177/13591053231151286 | OUTCOME |
| 168 | Singer S, Roick J, Meixensberger J, et al. The effects of multi-disciplinary psycho-social care on socio-economic problems in cancer patients: a cluster-randomized trial. Supportive care in cancer. 2018;26(6):1851‐1859. doi:10.1007/s00520-017-4024-x | AGE |
| 169 | Sjövall K, Attner B, Englund M, et al. Sickness absence among cancer patients in the pre-diagnostic and the post-diagnostic phases of five common forms of cancer. Supportive Care in Cancer. 2012;20(4):741-747. doi:10.1007/s00520-011-1142-8 | OUTCOME |
| 170 | Skaczkowski G, Asahina A, Wilson C. Returning to Work After Cancer in Australia: What Facilitates a Positive Return to Work Experience? Journal of Occupational Rehabilitation. 2021;31(1):41-49. doi:10.1007/s10926-020-09881-3 | OUTCOME |
| 171 | Söderman M, Friberg E, Alexanderson K, Wennman-Larsen A. Women’s experiences of encounters with healthcare professionals’ regarding work after breast-cancer surgery and associations with sickness absence: a 2-year follow-up cohort study. Support Care Cancer. 2019;27(4):1197-1206. doi:10.1007/s00520-018-4453-1 | OUTCOME |
| 172 | Sorrentino L, Fissi S, Meaglia I, et al. One-step intraoperative radiotherapy optimizes conservative treatment of breast cancer with advantages in quality of life and work resumption. Breast. 2018;39:123-130. doi:10.1016/j.breast.2018.04.004 | AGE |
| 173 | Sowden M, Vacek P, Geller BM. The impact of cancer diagnosis on employment: is there a difference between rural and urban populations? Journal of Cancer Survivorship. 2014;8(2):213-217. doi:10.1007/s11764-013-0317-3 | AGE |
| 174 | Stapelfeldt C, Momsen A, Jensen A, Andersen N, Nielsen C. Municipal return to work management in cancer survivors: a controlled intervention study. Acta oncologica (Stockholm, Sweden). 2021;60(3):370‐378. doi:10.1080/0284186X.2020.1853227 | OTHER |
| 175 | Stockdill ML, Mendoza T, Armstrong TS, Miaskowski C, Cooper B, Vera E. Identification of health-related quality of life profiles among long-term survivors of primary central nervous system tumors. J Neurooncol. 2023;165(1):181-190. doi:10.1007/s11060-023-04474-5 | OUTCOME |
| 176 | Streibelt M, Bethge M, Gross T, Herrmann K, Ustaoglu F, Reichel C. Predictive Validity of a Screening Instrument for the Risk of Non-Return to Work in Patients With Internal Diseases. Arch Phys Med Rehabil. 2017;98(5):989-996.e1. doi:10.1016/j.apmr.2016.12.022 | POP |
| 177 | Su M, Zhang N, Cai Y, et al. Work and income changes after cancer in rural China: A cross-sectional survey. Cancer Med. 2019;8(18):7859-7868. doi:10.1002/cam4.2627 | AGE |
| 178 | Su TT, Azzani M, Tan FL, Loh SY. Breast cancer survivors: return to work and wage loss in selected hospitals in Malaysia. Support Care Cancer. 2018;26(5):1617-1624. doi:10.1007/s00520-017-3987-y | AGE |
| 179 | Sveistrup J, Mortensen OS, Rosenschöld PM, Engelholm SA, Petersen PM. Employment and sick leave in patients with prostate cancer before, during and after radiotherapy. Scand J Urol. 2016;50(3):164-169. doi:10.3109/21681805.2015.1119190 | AGE |
| 180 | Svingel LS, Christensen SF, Kjærsgaard A, et al. Labor market affiliation of patients with myeloproliferative neoplasms: a population-based matched cohort study. Acta Oncologica. 2023;62(10):1286-1294. doi:10.1080/0284186X.2023.2251670 | AGE |
| 181 | Takahashi M, Tsuchiya M, Horio Y, et al. Job resignation after cancer diagnosis among working survivors in Japan: timing, reasons and change of information needs over time. Jpn J Clin Oncol. 2018;48(1):43-51. doi:10.1093/jjco/hyx143 | OUTCOME |
| 182 | Tamminga S, Jansen L, Frings-Dresen M, de Boer A. Long-term employment status and quality of life after cancer: a longitudinal prospective cohort study from diagnosis up to and including 5 years post diagnosis. Work (Reading, Mass). 2020;66(4):901‐907. doi:10.3233/WOR-203234 | OTHER |
| 183 | Tamminga S, Verbeek J, Frings-Dresen M, Boer A. Effectiveness of a hospital-based work support intervention for cancer patients-a multi-centre randomised controlled trial. Psycho-oncology. 2013;22:108‐. doi:10.1111/j.1099-1611.2013.3393 | OTHER |
| 184 | Tamminga SJ, Bültmann U, Husson O, Kuijpens JLP, Frings-Dresen MHW, de Boer AGEM. Employment and insurance outcomes and factors associated with employment among long-term thyroid cancer survivors: a population-based study from the PROFILES registry. Qual Life Res. 2016;25(4):997-1005. doi:10.1007/s11136-015-1135-z | OTHER |
| 185 | Tevaarwerk AJ, Kwekkeboom K, Buhr KA, et al. Results from a prospective longitudinal survey of employment and work outcomes in newly diagnosed cancer patients during and after curative-intent chemotherapy: A Wisconsin Oncology Network study. Cancer. 2021;127(5):801-808. doi:10.1002/cncr.33311 | POP |
| 186 | Tevaarwerk AJ, Lee JW, Sesto ME, et al. Employment outcomes among survivors of common cancers: the Symptom Outcomes and Practice Patterns (SOAPP) study. J Cancer Surviv. 2013;7(2):191-202. doi:10.1007/s11764-012-0258-2 | OTHER |
| 187 | Thaduri A, Garg PK, Malhotra M, et al. Financial toxicity and mental well-being of the oral cancer survivors residing in a developing country in the era of COVID 19 pandemic - A cross-sectional study. Psychooncology. 2023;32(1):58-67. doi:10.1002/pon.6030 | OUTCOME |
| 188 | Thielen K, Kolodziejczyk C, Andersen I, Heinesen E, Diderichsen F. Cancer stage, comorbidity, and socioeconomic differences in the effect of cancer on labour market participation: a danish register-based follow-up study. PLoS One. 2015;10(6):e0128621. doi:10.1371/journal.pone.0128621 | POP |
| 189 | Thijs K, Boer A, Vreugdenhil G, Wouw A, Houterman S, Schep G. Rehabilitation Using High-Intensity Physical Training and Long-Term Return-to-Work in Cancer Survivors. Journal of Occupational Rehabilitation. 2012;22(2):220-229. doi:10.1007/s10926-011-9341-1 | OTHER |
| 190 | Tiblom Ehrsson Y, Kisiel MA, Yang Y, Laurell G. Predicting Return to Work after Head and Neck Cancer Treatment Is Challenging Due to Factors That Affect Work Ability. Cancers. 2023;15(19):4705. doi:10.3390/cancers15194705 | OTHER |
| 191 | Tiedtke CM, Blonk RWB, Van Rhenen W, Van Egmond MP, Joosen MCW. Tailored support for preparing employees with cancer to return to work: Recognition and gaining new insights in an open atmosphere. Work. 2023;76(4):1519-1533. doi:10.3233/WOR-220566 | QUALITATIVE |
| 192 | Torp S, Nielsen RA, Gudbergsson SB, et al. Worksite adjustments and work ability among employed cancer survivors. Supportive Care in Cancer. 2012;20(9):2149-2156. doi:10.1007/s00520-011-1325-3 | OUTCOME |
| 193 | Torp S, Syse J, Paraponaris A, Gudbergsson S. Return to work among self-employed cancer survivors. J Cancer Surviv. 2017;11(2):189-200. doi:10.1007/s11764-016-0578-8 | POP |
| 194 | Trommer M, Marnitz S, Skoetz N, et al. Exercise interventions for adults with cancer receiving radiation therapy alone. Cochrane Database Syst Rev. 2023;3(3):CD013448. doi:10.1002/14651858.CD013448.pub2 | DESIGN |
| 195 | Tsuchiya M, Horio Y, Funazaki H, et al. Impact of gender and employment type on job loss among cancer survivors. Jpn J Clin Oncol. 2020;50(7):766-771. doi:10.1093/jjco/hyaa040 | POP |
| 196 | Ullrich A, Rath HM, Otto U, et al. Identifying Expectations of Delayed Return to Work in Patients with Prostate Cancer at the Beginning of a Cancer Rehabilitation Program. Journal of Occupational Rehabilitation. 2020;30(2):183-193. doi:10.1007/s10926-019-09860-3 | OUTCOME |
| 197 | Ullrich A, Rath HM, Otto U, et al. Outcomes across the return-to-work process in PC survivors attending a rehabilitation measure-results from a prospective study. Support Care Cancer. 2017;25(10):3007-3015. doi:10.1007/s00520-017-3790-9 | OUTCOME |
| 198 | Van Egmond M, Duijts S, Jonker M, Van Der Beek A, Anema J. Effectiveness of a return to work program for cancer survivors with job loss: results of a randomized controlled trial. Psycho-oncology. 2016;25:31‐32. doi:10.1002/pon.4272 | POP |
| 199 | van Muijen P, Duijts SF, Bonefaas-Groenewoud K, van der Beek AJ, Anema JR, Duijts SFA. Factors associated with work disability in employed cancer survivors at 24-month sick leave. BMC Cancer. 2014;14(1):236-236. doi:10.1186/1471-2407-14-236 | OUTCOME |
| 200 | van Muijen P, Duijts SFA, van der Beek AJ, Anema JR. Prognostic factors of work disability in sick-listed cancer survivors. Journal of Cancer Survivorship. 2013;7(4):582-591. doi:10.1007/s11764-013-0297-3 | OUTCOME |
| 201 | Vayr F, Montastruc M, Savall F, et al. Work adjustments and employment among breast cancer survivors: a French prospective study. Supportive Care in Cancer. 2020;28(1):185-192. doi:10.1007/s00520-019-04799-w | OUTCOME |
| 202 | von Mechow S, Graefen M, Haese A, et al. Return to work following robot-assisted laparoscopic and open retropubic radical prostatectomy: A single-center cohort study to compare duration of sick leave. Urol Oncol. 2018;36(6):309.e1-309.e6. doi:10.1016/j.urolonc.2018.02.006 | OTHER |
| 203 | Weaver KE, Geiger AM, Lu L, Case LD. Rural-urban disparities in health status among US cancer survivors. Cancer (0008543X). 2013;119(5):1050-1057. doi:10.1002/cncr.27840 | OUTCOME |
| 204 | Weaver KE, Palmer N, Lu L, Case LD, Geiger AM. Rural-urban differences in health behaviors and implications for health status among US cancer survivors. Cancer Causes Control. 2013;24(8):1481-1490. doi:10.1007/s10552-013-0225-x | AGE |
| 205 | Weemaes ATR, Beelen M, Weijenberg MP, van Kuijk SMJ, Lenssen AF. Effects of remote coaching following supervised exercise oncology rehabilitation on physical activity levels, physical fitness, and patient-reported outcomes: a randomised controlled trial. International Journal of Behavioral Nutrition & Physical Activity. 2024;21(1):1-15. doi:10.1186/s12966-024-01561-2 | OTHER |
| 206 | Wengström Y, Bolam K, Mijwel S, et al. Optitrain: a randomised controlled exercise trial for women with breast cancer undergoing chemotherapy. BMC cancer. 2017;17(1):100. doi:10.1186/s12885-017-3079-x | AGE |
| 207 | Weschenfelder W, Gast-Froehlich S, Spiegel C, Vogt M, Hofmann GO. Factors influencing quality of life, function, reintegration and participation after musculoskeletal tumour operations. BMC Cancer. 2020;20(1):351. doi:10.1186/s12885-020-06837-x | AGE |
| 208 | Wiemann G, Pertz M, Kowalski T, Seidel S, Schlegel U, Thoma P. Complete response to therapy: why do primary central nervous system lymphoma patients not return to work? J Neurooncol. 2020;149(1):171-179. doi:10.1007/s11060-020-03587-5 | AGE |
| 209 | Wolvers MDJ, Leensen MCJ, Groeneveld IF, Frings-Dresen MHW, De Boer AGEM. Predictors for earlier return to work of cancer patients. Journal of Cancer Survivorship. 2018;12(2):169-177. doi:10.1007/s11764-017-0655-7 | OTHER |
| 210 | Wong W, Dickerson JC, Valtis YK, et al. Cancer Demographics and Time-to-Care in Belize. Oncologist. 2023;28(6):e350-e358. doi:10.1093/oncolo/oyad030 | OUTCOME |
| 211 | Wood R, Mitra D, DeCourcy J, Iyer S. Work productivity and activity impairment in women with HR+/HER2-advanced/ metastatic breast cancer. Value in health Conference: ISPOR 22nd annual international meeting United states. 2017;20(5):A123. | CONFERENCE |
| 212 | Wu LM, Kuprian N, Herbert K, et al. A mixed methods analysis of perceived cognitive impairment in hematopoietic stem cell transplant survivors. Palliat Support Care. 2019;17(4):396-402. doi:10.1017/S1478951518000664 | QUALITATIVE |
| 213 | Yagil D, Cohen M. The role of perceived supervisor resistance in successful work sustainability among cancer survivors. Work. 2022;73(1):203-210. doi:10.3233/WOR-205341 | OUTCOME |
| 214 | Yoong W, Fadel MG, Walker S, Williams S, Subba B. Retrospective Cohort Study to Assess Outcomes, Cost-Effectiveness, and Patient Satisfaction in Primary Vaginal Ovarian Cystectomy versus the Laparoscopic Approach. J Minim Invasive Gynecol. 2016;23(2):252-256. doi:10.1016/j.jmig.2015.10.008 | OUTCOME |
| 215 | Yoshida A, Motomura K, Natsume A, et al. Preoperative predictive factors affecting return to work in patients with gliomas undergoing awake brain mapping. J Neurooncol. 2020;146(1):195-205. doi:10.1007/s11060-019-03371-0 | AGE |
| 216 | Yu J, Paranagama D, Geyer HL, Parasuraman S, Mesa R. Relationship between symptom burden and disability leave among patients with myeloproliferative neoplasms (MPNs): findings from the Living with MPN patient survey. Annals of Hematology. 2019;98(5):1119-1125. doi:10.1007/s00277-019-03610-4 | AGE |
| 217 | Yuan CM, Wang CC, Wu WT, Ho CL, Chen WL. Risk factors for return to work in colorectal cancer survivors. Cancer Med. 2021;10(12):3938-3951. doi:10.1002/cam4.3952 | AGE |
| 218 | Zaman A, Tytgat K, Klinkenbijl J, et al. Effectiveness of a Tailored Work-Related Support Intervention for Patients Diagnosed with Gastrointestinal Cancer: a Multicenter Randomized Controlled Trial. Journal of occupational rehabilitation. 2021;31(2):323‐338. doi:10.1007/s10926-020-09920-z | OTHER |
| 219 | Zapata Bonilla SA, Fried M, Singer S, et al. Working situation and burden of work limitations in sarcoma patients: results from the multi-center prospective PROSa study. J Cancer Res Clin Oncol. 2023;149(9):6009-6021. doi:10.1007/s00432-022-04556-3 | AGE |

***S2. Study evaluations with* *STrengthening the Reporting of OBservational studies in Epidemiology (STROBE).***

| **Quality assessment** | | | | | | | | | | | | | | | | | | | | | | | | |
| --- | --- | --- | --- | --- | --- | --- | --- | --- | --- | --- | --- | --- | --- | --- | --- | --- | --- | --- | --- | --- | --- | --- | --- | --- |
|  | **1** | **2** | **3** | **4** | **5** | **6** | **7** | **8** | **9** | **10** | **11** | **12** | **13** | **14** | **15** | **16** | **17** | **18** | **19** | **20** | **21** | **22** |  |  |
| First author | **Items from the STROBE checklist** | | | | | | | | | | | | | | | | | | | | | | **GRADE** | **Quality** |
| **Arndt (2019)** | 1 | 1 | 1 | $1$ | 1 | 1 | 1 | 1 | 1 | 1 | $0^{a}$ | 1 | $0^{b}$ | $0^{c}$ | 1 | 1 | 1 | 1 | 1 | 1 | 1 | 1 | **19** | **✓** |
| **Basalathullah (2021)** | 1 | 1 | 1 | 1 | 1 | 1 | $0^{d}$ | 1 | $0^{e}$ | 1 | 1 | $0^{f}$ | 1 | 1 | 1 | 1 | $0^{g}$ | 1 | 1 | 1 | 1 | 1 | **18** | **✓** |
| **Bennett (2018)** | 1 | 1 | 1 | 1 | 1 | 1 | 1 | 1 | 1 | 1 | 1 | $0^{h}$ | 1 | $1$ | 1 | $0^{i}$ | 1 | 1 | 1 | 1 | 1 | 1 | **20** | **✓** |
| **Blinder (2012)** | 1 | 1 | 1 | 1 | 1 | 1 | 1 | 1 | 1 | 1 | 1 | 1 | 1 | $0^{j}$ | 1 | 1 | 1 | 1 | 1 | 1 | 1 | 1 | **21** | **✓** |
| **Caumette (2021)** | 1 | 1 | 1 | 1 | 1 | 1 | 1 | 1 | 1 | 1 | 1 | 1 | 1 | 1 | 1 | 1 | 1 | 1 | 1 | 1 | $0^{k}$ | 1 | **21** | **✓** |
| **Check (2019)** | $0^{l}$ | 1 | 1 | 1 | 1 | 1 | 1 | 1 | $0^{m}$ | 1 | 1 | $0^{n}$ | $0^{o}$ | $0^{p}$ | 1 | 1 | 1 | 1 | 1 | 1 | 1 | 1 | **17** | **X** |
| **Chen S (2019)** | 1 | 1 | 1 | 1 | 1 | 1 | $0^{q}$ | 1 | $0^{r}$ | 1 | 1 | $0^{s}$ | 1 | 1 | 1 | 1 | 1 | 1 | 1 | 1 | 1 | 1 | **19** | **✓** |
| **Chen W (2021)** | 1 | 1 | 1 | 1 | 1 | 1 | 1 | 1 | $0^{t}$ | 1 | 1 | $0^{u}$ | 1 | $0^{v}$ | 1 | 1 | 1 | 1 | 1 | 1 | 1 | 1 | **19** | **✓** |
| **Chen Y (2020)** | 1 | 1 | 1 | 1 | 1 | 1 | 1 | $1$ | $0^{w}$ | 1 | 1 | $0^{x}$ | 1 | 1 | 1 | 1 | 1 | 1 | 1 | 1 | 1 | 1 | **20** | **✓** |
| **Chiu (2023)** | 1 | 1 | 1 | 1 | 1 | 1 | 1 | 1 | 1 | 1 | 1 | $0^{y}$ | $0^{z}$ | $0^{aa}$ | 1 | 1 | 1 | 1 | 1 | 1 | 1 | 1 | **19** | **✓** |
| **Colombino (2020)** | 1 | 1 | $1$ | 1 | 1 | 1 | $1$ | $0^{ab}$ | $0^{ac}$ | 1 | 1 | 1 | 1 | 1 | 1 | $0^{ad}$ | 1 | 1 | 1 | 1 | 1 | $0^{ae}$ | **18** | **✓** |
| **Dahl (2014)** | 1 | 1 | 1 | 1 | 1 | 1 | 1 | 1 | $0^{af}$ | $0^{ag}$ | 1 | $0^{ah}$ | 1 | $0^{ai}$ | 1 | $0^{aj}$ | 1 | 1 | 1 | 1 | 1 | 1 | **17** | **X** |
| **den Bakker (2020)** | 1 | 1 | 1 | 1 | 1 | 1 | 1 | 1 | 1 | $0^{ak}$ | 1 | 1 | 1 | 1 | 1 | 1 | 1 | 1 | 1 | 1 | 1 | 1 | **21** | **✓** |
| **Dumas (2020)** | 1 | 1 | 1 | 1 | 1 | $1$ | 1 | 1 | $1$ | 1 | 1 | 1 | 1 | 1 | 1 | 1 | 1 | 1 | 1 | 1 | 1 | 1 | **22** | **✓** |
| **Endo (2016)** | 1 | 1 | 1 | 1 | 1 | 1 | 1 | 1 | 1 | 1 | 1 | 1 | 1 | 1 | 1 | 1 | 1 | 1 | 1 | 1 | 1 | $0^{al}$ | **21** | **✓** |
| **Fiabane (2024)** | 1 | 1 | 1 | 1 | 1 | 1 | 1 | 1 | 1 | $0^{am}$ | 1 | $0^{an}$ | 1 | 1 | 1 | 1 | 1 | 1 | $0^{ao}$ | 1 | 1 | 1 | **19** | **✓** |
| **Gordon (2014)** | 1 | 1 | 1 | 1 | 1 | 1 | 1 | 1 | 1 | 1 | 1 | 1 | 1 | 1 | 1 | $0^{ap}$ | 1 | 1 | 1 | 1 | 1 | 1 | **21** | **✓** |
| **Hedayati (2013)** | $0^{aq}$ | 1 | $1$ | $1$ | $1$ | 1 | 1 | 1 | 1 | $0^{ar}$ | 1 | 1 | 1 | 1 | 1 | 1 | 1 | 1 | 1 | 1 | 1 | 1 | **20** | **✓** |
| **Horsboel (2015)** | 1 | 1 | 1 | 1 | 1 | 1 | 1 | 1 | 1 | $0^{as}$ | 1 | 1 | 1 | 1 | 1 | 1 | 1 | 1 | 1 | 1 | 1 | 1 | **21** | **✓** |
| **Horsboel (2013)** | 1 | 1 | 1 | 1 | 1 | 1 | 1 | 1 | 1 | 1 | 1 | 1 | 1 | 1 | 1 | 1 | 1 | 1 | 1 | 1 | 1 | 1 | **22** | **✓** |
| **Johnsson (2024)** | 1 | 1 | 1 | 1 | 1 | 1 | 1 | 1 | 1 | $0^{at}$ | 1 | $0^{au}$ | 1 | 1 | 1 | 1 | 1 | 1 | 1 | 1 | 1 | 1 | **20** | **✓** |
| **Ito (2015)** | $0^{av}$ | 1 | 1 | 1 | 1 | 1 | 1 | 1 | $0^{aw}$ | $0^{ax}$ | 1 | $0^{ay}$ | 1 | 1 | 1 | $0^{az}$ | 1 | 1 | 1 | 1 | $0^{ba}$ | $0^{bb}$ | **15** | **X** |
| **Kang (2022)** | 1 | 1 | 1 | 1 | 1 | 1 | 1 | 1 | $1$ | $0^{bc}$ | 1 | $0^{bd}$ | $0^{be}$ | 1 | 1 | 1 | 1 | 1 | 1 | 1 | 1 | 1 | **19** | **✓** |
| **Landeiro (2018)** | 1 | 1 | 1 | 1 | 1 | 1 | 1 | 1 | 1 | 1 | 1 | 1 | 1 | 1 | 1 | 1 | 1 | 1 | 1 | 1 | 1 | 1 | **22** | **✓** |
| **Li (2021)** | $0^{bf}$ | 1 | 1 | 1 | 1 | 1 | 1 | 1 | 1 | $0^{bg}$ | 1 | $0^{bh}$ | 1 | 1 | 1 | 1 | 1 | 1 | 1 | 1 | 1 | 1 | **19** | **✓** |
| **Lindbohm (2014)** | $0^{bi}$ | 1 | 1 | 1 | 1 | 1 | 1 | 1 | 1 | 1 | 1 | 1 | 1 | $0^{bj}$ | $1$ | 1 | 1 | 1 | 1 | 1 | $0^{bk}$ | 1 | **19** | **✓** |
| **Morales (2020)** | 1 | 1 | 1 | 1 | 1 | 1 | $0^{bl}$ | $0^{bm}$ | $0^{bn}$ | $0^{bo}$ | 1 | 1 | 1 | 1 | 1 | 1 | 1 | 1 | 1 | 1 | 1 | 1 | **18** | **✓** |
| **Paltrinieri (2022)** | 1 | 1 | 1 | 1 | 1 | 1 | 1 | 1 | $1$ | $0^{bp}$ | 1 | $0^{bq}$ | 1 | 1 | 1 | 1 | 1 | 1 | 1 | 1 | 1 | 1 | **20** | **✓** |
| **Paltrinieri (2020)** | 1 | 1 | 1 | 1 | 1 | 1 | 1 | 1 | 1 | $0^{br}$ | 1 | $0^{bs}$ | 1 | 1 | 1 | 1 | 1 | 1 | 1 | 1 | 1 | 1 | **20** | **✓** |
| **Rashid (2021)** | 1 | 1 | 1 | 1 | 1 | 1 | 1 | 1 | 1 | $0^{bt}$ | 1 | 1 | 1 | 1 | 1 | 1 | 1 | 1 | 1 | 1 | 1 | 1 | **21** | **✓** |
| **Rosbjerg (2020)** | 1 | 1 | 1 | 1 | 1 | 1 | 1 | 1 | 1 | $0^{bu}$ | 1 | 1 | 1 | 1 | 1 | 1 | 1 | 1 | 1 | 1 | 1 | 1 | **21** | **✓** |
| **Ross (2012)** | 1 | 1 | 1 | 1 | 1 | 1 | 1 | 1 | 1 | $0^{bv}$ | 1 | $0^{bw}$ | 1 | 1 | 1 | 1 | 1 | $1$ | 1 | 1 | 1 | $0^{bx}$ | **19** | **✓** |
| **Rydén (2020)** | 1 | 1 | 1 | 1 | 1 | 1 | 1 | 1 | 1 | 1 | 1 | $0^{by}$ | 1 | 1 | 1 | 1 | 1 | $1$ | 1 | 1 | 1 | 1 | **21** | **✓** |
| **Schmidt (2019)** | $0^{bz}$ | 1 | 1 | 1 | 1 | 1 | 1 | 1 | 1 | $0^{ca}$ | $0^{cb}$ | $0^{cc}$ | 1 | 1 | 1 | 1 | 1 | 1 | 1 | 1 | 1 | $0^{cd}$ | **17** | **X** |
| **Sesto (2022)** | 1 | 1 | 1 | 1 | 1 | 1 | $0^{ce}$ | $0^{cf}$ | $0^{cg}$ | $0^{ch}$ | $1$ | $0^{ci}$ | 1 | $0^{cj}$ | 1 | 1 | 1 | 1 | 1 | 1 | 1 | 1 | **16** | **X** |
| **So (2020)** | $0^{ck}$ | 1 | 1 | 1 | 1 | 1 | 1 | 1 | 1 | $0^{cl}$ | 1 | 1 | 1 | 1 | 1 | 1 | $1$ | 1 | 1 | 1 | 1 | $0^{cm}$ | **19** | **✓** |
| **Starnoni (2018)** | 1 | 1 | 1 | 1 | 1 | 1 | 1 | 1 | 1 | $0^{cn}$ |  | 1 | 1 | 1 | 1 | 1 | 1 | 1 | 1 | 1 | $0^{co}$ | $0^{cp}$ | **19** | **✓** |
| **Ullrich (2017)** | 1 | 1 | 1 | 1 | 1 | 1 | 1 | 1 | 1 | $0^{cq}$ | 1 | $0^{cr}$ | 1 | 1 | 1 | $1$ | 1 | 1 | 1 | 1 | 1 | 1 | **20** | **✓** |
| **Ullrich (2018)** | 1 | 1 | 1 | 1 | 1 | 1 | 1 | 1 | $1$ | 1 | 1 | 1 | 1 | 1 | 1 | 1 | 1 | 1 | 1 | 1 | 1 | 1 | **22** | **✓** |
| **Ullrich (2022)** | 1 | 1 | 1 | 1 | 1 | 1 | 1 | 1 | $1$ | 1 | 1 | 1 | 1 | 1 | 1 | 1 | 1 | 1 | 1 | 1 | 1 | 1 | **22** | **✓** |
| **van Loon (2021)** | 1 | 1 | 1 | 1 | 1 | 1 | 1 | 1 | $0^{cs}$ | $0^{ct}$ | 1 | $0^{cu}$ | 1 | $0^{cv}$ | 1 | 1 | 1 | 1 | 1 | 1 | $0^{cw}$ | $0^{cx}$ | **16** | **X** |
| **Yang S (2021)** | 1 | 1 | 1 | 1 | 1 | 1 | 1 | 1 | $1$ | $1$ | $1$ | $0^{cy}$ | 1 | 1 | 1 | 1 | 1 | 1 | 1 | 1 | 1 | 1 | **21** | **✓** |
| **Yang Z (2021)** | 1 | 1 | 1 | 1 | 1 | 1 | 1 | 1 | $1$ | 1 | 1 | 1 | 1 | 1 | 1 | 1 | 1 | 1 | 1 | 1 | 1 | 1 | **22** | **✓** |

a: No explanation of how quantitative variables were reported.

b: No reasons for non-participation at each stage were reported.

c: No missing data were reported.

d: Potential confounders or effect modifiers weren’t clearly defined.

e: No efforts to address potential sources of bias were found.

f: No description of statistical methods to control for confounding and no explanation of how missing data were reported.

g: Other analyses conducted weren’t reported.

h: No explanations of how missing data were addressed.

i: Adjustments for confounding were not reported.

j: Number of participants with missing data was not reported.

k: External validity was not discussed.

l: Study design was not indicated in the title and abstract with a commonly used term.

m: No efforts to address potential sources of bias were found.

n: No explanations of how missing data were addressed.

o: No reasons for non-participation at each stage were reported, flow diagram was not used.

p: Number of participants with missing data was not reported.

q: Predictors, potential confounders, and diagnostic criteria were not discussed.

r: No efforts to address potential sources of bias were found.

s: Interactions were not explored. No explanation of the treatment of missing data was reported.

t: No efforts to address potential sources of bias were found.

u: No explanations of how missing data were addressed were found.

v: Number of participants with missing data was not reported.

w: No efforts to address potential sources of bias were found.

x: No explanations of how missing data were addressed were found.

y: No explanations of how missing data were addressed were found.

z: Number of exclusions was not reported.

aa: Number of participants with missing data was not reported.

ab: Methods of measurement of the outcome (RTW) were not reported.

ac: No efforts to address potential sources of bias were found.

ad: Adjustments for confounding control were not reported.

ae: The source of funding was not found in the paper.

af: No efforts to address potential sources of bias were found.

ag: How the sample size was arrived at was not explained.

ah: No explanations of how missing data were addressed were found.

ai: Number of participants with missing data was not reported.

aj: Adjustments for confounding control were not clearly defined.

ak: The explanations of the study size was arrived at were not reported.

al: The source of funding was not found in the paper.

am: The explanations of the study size was arrived at were not reported.

an: missing data were not reported.

ao: The magnitude of any potential bias was not discussed.

ap: It was not clear for which variables the model was adjusted and why.

aq: Study design was not reported in title and abstract with a commonly used terms.

ar: How the sample size was arrived at was not explained.

as: How the sample size was arrived at was not explained.

at: How the sample size was arrived at was not explained.

au: No explanations of how missing data were addressed were found.

av: Study design was not specified in the title and abstract with a commonly used term.

aw: No efforts to address potential sources of bias were found.

ax: How the sample size was arrived at was not explained.

ay: How missing data was addressed was not explained.

az: The model’s adjustments were not clear.

ba: External validity was not discussed.

bb: The source of funding was not found in the paper.

bc: How the sample size was arrived at was not explained.

bd: Missing data were not reported.

be: Numbers of individuals were not reported at each stage of the study.

bf: Study design was not indicated in the title and abstract with a commonly used term.

bg: How the sample size was arrived at was not explained.

bh: Missing data were not reported.

bi: Study design was not indicated in the title and abstract with a commonly used term.

bj: Missing data were not reported.

bk: External validity was not discussed.

bl: The definition of occupation (before and after events) were not clearly defined.

bm: Methods of measurement of the outcome were not clearly defined.

bn: No efforts to address potential sources of bias were found.

bo: How the sample size was arrived at was not explained.

bp: How the sample size was arrived at was not explained.

bq: Any explanations of how missing data were addressed were not found.

br: How the sample size was arrived at was not explained.

bs: Any explanations of how missing data were addressed were not found.

bt: How the sample size was arrived at was not explained.

bu: How the sample size was arrived at was not explained.

bv: How the sample size was arrived at was not explained.

bw: Any explanations of how missing data were addressed were not found.

bx: The source of funding was not found in the paper.

by: Any explanations of how missing data were addressed were not found.

bz: Study design was not indicated in the title and abstract with a commonly used term.

ca: How the sample size was arrived at was not explained.

cb: Quantitative variables (Arm problems) were not sufficiently described of how it was handled.

cc: Any explanations of how missing data were addressed were not found.

cd: The source of funding was not found in the paper.

ce: The information collected was not clearly defined.

cf: Methods of measurement of the variables were not clearly defined.

cg: No efforts to address potential sources of bias were described.

ch: How the sample size was arrived at was not explained.

ci: Any explanations of how missing data were addressed were not found.

cj: Missing data were not reported.

ck: Study design was not indicated in the title and abstract with a commonly used term.

cl: How the sample size was arrived at was not explained.

cm: The source of funding was not found in the paper.

cn: How the sample size was arrived at was not explained.

co: External validity was not discussed.

cp: The source of funding was not found in the paper.

cq: How the sample size was arrived at was not explained.

cr: No explanations of how missing data were addressed were found.

cs: No efforts to address potential sources of bias were found.

ct: How the sample size was arrived at was not explained.

cu: No explanations of how missing data were addressed were found.

cv: Missing data were not reported.

cw: External validity was not discussed.

cx: The source of funding was not found in the paper.

cy: No explanations of how missing data were addressed were found.

***S3. Study evaluations with* *Risk of Bias tool* *(RoB2).***

| **Aim of the study** | **Author (year)** | **D1 Randomisation process** | **D2**  **Deviations from the intended interventions** | **D3**  **Missing outcome data** | **D4 Measurement of the outcome** | **D5**  **Selection of the reported result** | **Overall** | \| Low risk; \| High risk; \| Some concerns |
| --- | --- | --- | --- | --- | --- | --- | --- | --- |
| Intention-to-treat | Tamminga S. (2019) |  |  |  |  |  |  |  |

***S4. Study evaluations with* *Grading of Recommendations, Assessment, Development, and Evaluations (GRADE).***

|  | Failure to develop and apply appropriate eligibility criteria (inclusion of control population) | Under- or over-matching in case-control studies | Selection of exposed and unexposed in cohort studies from different populations | Flawed measurement of both exposure and outcome | Differences in measurement of exposure (e.g. recall bias in case-control studies) | Differential surveillance for outcome in exposed and unexposed in cohort studies | Failure to adequately control confounding | Failure to match for prognostic factors and/or adjustment in statistical analysis | Failure of accurate measurement of all known prognostic factors | Incomplete or inadequately short follow-up | ***OVERALL RESULTS*** |
| --- | --- | --- | --- | --- | --- | --- | --- | --- | --- | --- | --- |
| First authors |  |  |  |  |  |  |  |  |  |  |  |
| **Arndt (2019)** |  |  | ● |  |  | ● |  |  | ●${}^{1}$ | ● | ● |
| **Basalathullah (2021)** |  |  | ●${}^{2}$ |  |  | ●${{}^{3}}$ |  |  | ●${}^{4}$ | ● | ● |
| **Bennett (2018)** |  |  | ● |  |  | ● |  |  | ●${}^{5}$ | ● | ● |
| **Blinder (2012)** |  |  | ●${}^{6}$ |  |  | ● |  |  | ● | ● | ● |
| **Caumette (2021)** |  |  | ● |  |  | ● |  |  | ● | ● | ● |
| **Chen W (2021)** |  |  | ● |  |  | ● |  |  | ● | ● | ● |
| **Chen Y (2020)** |  |  | ●${}^{7}$ |  |  | ● |  |  | ● | ●${}^{8}$ | ● |
| **Dahl (2014)** |  |  | ● |  |  | ● |  |  | ● | ●${}^{9}$ | ● |
| **den Bakker (2020)** |  |  | ● |  |  | ● |  |  | ● | ●${}^{10}$ | ● |
| **Dumas (2020)** |  |  | ● |  |  | ●${}^{11}$ |  |  | ● | ●${}^{12}$ | ● |
| **Endo (2016)** |  |  | ● |  |  | ● |  |  | ● | ●${}^{13}$ | ● |
| **Fiabane (2024)** |  |  | ● |  |  | ● |  |  | ● | ●${}^{14}$ | ● |
| **Gordon (2014)** |  |  | ● |  |  | ● |  |  | ● | ●${}^{15}$ | ● |
| **Hedayati (2013)** |  |  | ● |  |  | ● |  |  | ● | ●${}^{16}$ | ● |
| **Horsboel (2015)** |  |  | ● |  |  | ● |  |  | ● | ●${}^{17}$ | ● |
| **Horsboel (2013)** |  |  | ● |  |  | ● |  |  | ● | ● | ● |
| **Johnsson (2023)** |  |  | ● |  |  | ● |  |  | ● | ●${}^{18}$ | ● |
| **Landeiro (2018)** |  |  | ● |  |  | ● |  |  | ●${}^{19}$ | ●${}^{20}$ | ● |
| **Li (2021)** |  |  | ● |  |  | ● |  |  | ● | ●${}^{21}$ | ● |
| **Rosbjerg (2020)** |  |  | ● |  |  | ● |  |  | ● | ●${}^{22}$ | ● |
| **Rydén (2020)** |  |  | ● |  |  | ● |  |  | ● | ●${}^{23}$ | ● |
| **Schmidt (2019)** |  |  | ● |  |  | ● |  |  | ● | ● | ● |
| **Starnoni (2018)** |  | ● |  |  | ●${}^{24}$ |  |  | ●${}^{25}$ |  | ●${}^{26}$ | ● |
| **Ullrich (2017)** |  |  | ● |  |  | ● |  |  | ●${}^{27}$ | ●${}^{28}$ | ● |
| **Ullrich (2018)** |  |  | ● |  |  | ● |  |  | ● | ●${}^{29}$ | ● |
| **Ullrich (2022)** |  |  | ● |  |  | ● |  |  | ● | ●${}^{30}$ | ● |
| **Yang SW (2021)** |  |  | ● |  |  | ● |  |  | ● | ● | ● |
| **Yang ZY (2021)** |  |  | ● |  |  | ● |  |  | ● | ● | ● |

1: Residual confounding as in every observational study, which is susceptible to confounding by indication, channeling bias and many other unmeasured or unreported confounding factors

2: Subject are selected as consecutive brain tumor patients treated during the study period. This type of selection may introduce bias or difficulties for the external validity.

3: Due to the retrospective and cross-sectional nature there are several limitations in this study, such as recall and time bias.

4: The most important problem in this paper was the unreported effects measures.

5 : Residual confounding as in every observational study, which is susceptible to confounding by indication, channeling bias and many other unmeasured or unreported confounding factors

6 : Selection bias due to the study design: selection included only uninsured/underinsured and below-poverty-level participants.

7: Does not give info on how the unexposed population was recruited.

8: 24-month follow-up.

9: 24 month follow-up.

10: 12-24 month follow-up.

11: Does not give info on how the RTW data were collected.

12: 12-24 month follow-up.

13: 12 month follow-up.

14: 6-month follow-up.

15: 6-12 month follow-up.

16: 18 month follow-up.

17: 12 month follow-up.

18: 1 year follow-up.

19: Does not report effect sizes of each covariate considered.

20: 6-12-24 month follow-up.

21: 31 month follow-up.

22: 15 month follow-up.

23: 1 and 2 years follow-up.

24: The exact date (month and day) related to the resumption of work, as well as its precise duration, was not precisely reported.

25: The most important problem in this paper was the unreported effects measures.

26: 6,12,18,24 month follow-up.

27: Unreported effects measures.

28: 12 month follow-up.

29: 12 month follow-up.

30: 12 month follow-up.

***S5. Forest plot and funnel plot for Age (≥50 vs <50) (RTW outcome).***

***S6. Forest plot and funnel plot for Sex (Female vs Male) (RTW outcome).***

***S7. Forest plot and funnel plot for Cohabiting (yes vs no) (RTW outcome).***

***S8. Forest plot and funnel plot for Educational level (Low vs Medium-High) (RTW outcome).***

***S9. Forest plot and funnel plot for Income (Low vs Medium-High) (RTW outcome).***

***S10. Forest plot and funnel plot for Type of contract (part- vs full-time) (RTW outcome).***

***S11. Forest plot and funnel plot for Self-employment (yes vs no) (RTW outcome).***

***S12. Forest plot and funnel plot for Designation of workers (Blue- vs White-collar) (RTW outcome).***

***S13. Forest plot and funnel plot for Clinical stage (2+ vs 0-1) (RTW outcome).***

***S14. Forest plot and funnel plot for Comorbidities (1+ vs 0) (RTW outcome).***

***S15. Forest plot and funnel plot for Radiotherapy (yes vs no) (RTW outcome).***

***S16. Forest plot and funnel plot for Chemotherapy (yes vs no) (RTW outcome).***

***S17. Forest plot and funnel plot for Surgery (yes vs no) (RTW outcome).***

***S18. Forest plot and funnel plot for Sex (Female vs Male) (Time To RTW outcome).***

***S19. Forest plot and funnel plot for Radiotherapy (yes vs no) (Time To RTW outcome).***

***S20. Forest plot and funnel plot for Chemotherapy (yes vs no) (Time To RTW outcome).***

***S21. Egger test and trim-and-fill analysis by Exposure for RTW outcome.***

| **AGE ≥50** | | | |
| --- | --- | --- | --- |
| **Studies** | **Effect size** | **95% CI** | **Egger test** |
| **Observed** | **-0.604** | **-0.910 to -0.299** | **Prob > \|z\| = 0.0154** |
| **Observed + Imputed** | **-0.604** | **-0.910 to -0.299** |  |
| **SEX** | | | |
| **Studies** | **Effect size** | **95% CI** | **Egger test** |
| **Observed** | **-0.117** | **-0.427 to 0.193** | **Prob > \|z\| = 0.4010** |
| **Observed + Imputed** | **0.038** | **-0.309 to 0.385** |  |
| **COHABITING** | | | |
| **Studies** | **Effect size** | **95% CI** | **Egger test** |
| **Observed** | **0.007** | **-0.238 to 0.252** | **Prob > \|z\| = 0.6817** |
| **Observed + Imputed** | **0.038** | **-0.210 to 0.285** |  |
| **EDUCATIONAL LEVEL** | | | |
| **Studies** | **Effect size** | **95% CI** | **Egger test** |
| **Observed** | **-0.569** | **-0.865 to -0.273** | **Prob > \|z\| = 0.8011** |
| **Observed + Imputed** | **-0.569** | **-0.865 to -0.273** |  |
| **INCOME** | | | |
| **Studies** | **Effect size** | **95% CI** | **Egger test** |
| **Observed** | **-0.315** | **-0.776 to 0.147** | **Prob > \|z\| = 0.0709** |
| **Observed + Imputed** | **-0.315** | **-0.776 to 0.147** |  |
| **TYPE OF CONTRACT** | | | |
| **Studies** | **Effect size** | **95% CI** | **Egger test** |
| **Observed** | **-0.114** | **-0.503 to 0.274** | **Prob > \|z\| = 0.0658** |
| **Observed + Imputed** | **0.005** | **-0.361 to 0.372** |  |
| **SELF-EMPLOYMENT** | | | |
| **Studies** | **Effect size** | **95% CI** | **Egger test** |
| **Observed** | **0.193** | **-0.164 to 0.549** | **Prob > \|z\| = 0.0351** |
| **Observed + Imputed** | **0.193** | **-0.164 to 0.549** |  |
| **DESIGNATION OF WORKERS** | | | |
| **Studies** | **Effect size** | **95% CI** | **Egger test** |
| **Observed** | **-0.407** | **-0.668 to -0.146** | **Prob > \|z\| = 0.0731** |
| **Observed + Imputed** | **-0.277** | **-0.592 to 0.038** |  |
| **CLINICAL STAGE** | | | |
| **Studies** | **Effect size** | **95% CI** | **Egger test** |
| **Observed** | **-0.612** | **-0.809 to -0.414** | **Prob > \|z\| = 0.0937** |
| **Observed + Imputed** | **-0.724** | **-0.934 to -0.514** |  |
| **COMORBIDITIES** | | | |
| **Studies** | **Effect size** | **95% CI** | **Egger test** |
| **Observed** | **-0.595** | **-1.216 to 0.027** | **Prob > \|z\| = 0.8448** |
| **Observed + Imputed** | **-0.595** | **-1.216 to 0.027** |  |
| **RADIOTHERAPY** | | | |
| **Studies** | **Effect size** | **95% CI** | **Egger test** |
| **Observed** | **-0.367** | **-0.631 to -0.103** | **Prob > \|z\| = 0.4777** |
| **Observed + Imputed** | **-0.422** | **-0.683 to -0.160** |  |
| **CHEMOTHERAPY** | | | |
| **Studies** | **Effect size** | **95% CI** | **Egger test** |
| **Observed** | **-0.483** | **-0.701 to -0.264** | **Prob > \|z\| = 0.0473** |
| **Observed + Imputed** | **-0.483** | **-0.701 to -0.264** |  |
| **SURGERY** | | | |
| **Studies** | **Effect size** | **95% CI** | **Egger test** |
| **Observed** | **0.037** | **-0.452 to 0.527** | **Prob > \|z\| = 0.0469** |
| **Observed + Imputed** | **0.110** | **-0.371 to 0.592** |  |

***S22. Egger test and trim-and-fill analysis by Exposure for Time To RTW outcome.***

| **SEX** | | | |
| --- | --- | --- | --- |
| **Studies** | **Effect size** | **95% CI** | **Egger test** |
| **Observed** | **-0.179** | **-0.296 to -0.061** | **Prob > \|z\| = 0.6675** |
| **Observed + Imputed** | **-0.134** | **-0.264 to -0.003** |  |
| **RADIOTHERAPY** | | | |
| **Studies** | **Effect size** | **95% CI** | **Egger test** |
| **Observed** | **-0.079** | **-0.278 to 0.121** | **Prob > \|z\| = 0.3117** |
| **Observed + Imputed** | **0.032** | **-0.171 to 0.236** |  |
| **CHEMOTHERAPY** | | | |
| **Studies** | **Effect size** | **95% CI** | **Egger test** |
| **Observed** | **-0.055** | **-0.209 to 0.100** | **Prob > \|z\| = 0.6286** |
| **Observed + Imputed** | **-0.055** | **-0.209 to 0.100** |  |

***S23. Forest and funnel plots from Sensitivity Analyses of Age (≥50 vs <50) exposure for the RTW outcome.***

***S24. Forest and funnel plots from Sensitivity Analyses of Sex (Female vs Male) exposure for the RTW outcome.***

***S25. Forest and funnel plots from Sensitivity Analyses of Cohabiting (yes vs no) exposure for the RTW outcome.***

***S26. Forest and funnel plots from Sensitivity Analyses of Educational level (Low vs Medium-High) exposure for the RTW outcome.***

***S27. Forest and funnel plots from Sensitivity Analyses of Income (Low vs Medium-High) exposure for the RTW outcome.***

***S28. Forest and funnel plots from Sensitivity Analyses of Type of contract (part- vs full-time) exposure for the RTW outcome.***

***S29. Forest and funnel plots from Sensitivity Analyses of Self-employment (yes vs no) exposure for the RTW outcome.***

***S30. Forest and funnel plots from Sensitivity Analyses of Designation of workers (Blue- vs White-collar) exposure for the RTW outcome.***

***S31. Forest and funnel plots from Sensitivity Analyses of Clinical stage (2+ vs 0-1) exposure for the RTW outcome.***

***S32. Forest and funnel plots from Sensitivity Analyses of Radiotherapy (yes vs no) exposure for the RTW outcome.***

***S33. Forest and funnel plots from Sensitivity Analyses of Chemotherapy (yes vs no) exposure for the RTW outcome.***

***S34. Forest and funnel plots from Sensitivity Analyses of Surgery (yes vs no) exposure for the RTW outcome.***

***S35. Forest and funnel plots from Sensitivity Analyses of Sex (Female vs Male) exposure for the RTW outcome (sex-specific cancer excluded).***

***S36. Forest and funnel plots from Sensitivity Analyses of Sex (Female vs Male) exposure for Time to RTW outcome (sex-specific cancer excluded).***

***S37. Motivations for assigning ‘some concern’ and ‘high risk’ ratings to items in each observational study.***

| **Cohort studies** | |
| --- | --- |
| Arndt (2019) | 1. The study **does not include an unexposed cohor.** 2. The occupational outcome was detected by means of **a self-reported postal questionnaire.** 3. Of the **14,526 eligible survivors**, only **6,057 (42%)** returned the full questionnaire; among working-age survivors, the rate was **44%.** In addition, the study includes only subjects who were **alive at the time of the survey** and able to respond, with potential important selection bias recognized by the authors. |
| Basalathullah (2021) | 1. The cohort is derived from **a single tertiary center** and is very selected. 2. There is no **unexposed cohort.** 3. The authors report mainly **descriptive analyses and univariate associations with chi-square;** there is no true **multivariable model** to control for the main confounders. 4. Data on work and return to work were collected by **questionnaire/interview**, during follow-up visits or **by telephone**, then by **self-report**. 5. The flow of participants is described, but the final cohort is small and highly selected: from **125 patients evaluated**, many were excluded; in addition, **2 non-contactable**, **1 refusal**, **4 deaths excluded** from the final analysis |
| Bennett (2018) | 1. The employment outcome is detected through a **self-report postal questionnaire**. |
| Blinder (2012) | 2. The authors perform a multivariable logistic regression to identify independent correlates of occupational status at 18 months. However, the control of confounders does not appear as extensive or systematic as in more robust studies; moreover, not all key factors are clearly included in a single predefined theoretical model. 3. The main outcome, i.e. **employment status**, is based on **self-reports** through telephone interviews. |
| Coumette (2021) | 1. The main exposures were collected via **self-report questionnaire at diagnosis**. 2. The 2-year RTW was collected via **self-report questionnaire.** 3. A significant proportion is excluded due to **RTW information missing at 2 years (n=717),** as well as relapses, withdrawals of consent, losses at follow-up and deaths. |
| Dumas (2020) | 1. Information on **outcome were collected via** **self-reported paper questionnaires.** 2. Although attrition is described, the non-response rate is not negligible and can introduce selection bias. |
| Endo (2016) | 1. Analyses were by **age, sex, and tumor site**. This indicates some confounding control. However, the study does not have fundamental clinical variables such as **stage of disease, tumor grade, type of treatment and comorbidities**, which the authors themselves acknowledge as missing. |
| Fiabane (2024) | 1. The sample comes from **a single cancer center.** 2. The main exposures (psychological factors, RTW expectations, QoL, work stress, resilience) are collected through **self-report questionnaires**. 3. Adjustements are limited: it only includes significant variables in preliminary analysis and does not systematically cover all potential clinical and socioeconomic confounders. 4. The outcome was assessed via **telephone self-report.** |
| Gordon (2014) | 1. The outcome was collected through **telephone interviews and self-report questionnaires**. |
| Hedayati (2013) | 1. The sample was small and selected from **a single hospital.** 2. There is no true **unexposed comparison cohort.** 3. The authors use statistical models (generalized linear models) adjusting for **age, education, marital status and work status baseline**. However, confounding control is limited, and the small sample prevents robust models. |
| Horsboel (2015) | 1. There is no true **unexposed cohort.** |
| Horsboel (2013) | 1. There is no true **unexposed cohort.** |
| Johnsson (2023) | 1. There is no unexposed cohort. |
| Landeiro (2018) | 1. The sample was single-center with a low participation rate. 2. There is no unexposed cohort. 3. Absence of a complete theoretical model, possible residual confounding. 4. RTW is **self-reported via telephone interviews.** |
| Li (2021) | 1. The cohort was monocentric and selected. 2. There is no unexposed cohort. 3. RTW is assessed via **telephone interview (self-report).** |
| Rosbjerg (2020) | 1. No true unexposed group. |
| Rydén (2020) | 1. The cohort was selected. |
| Schmidt (2019) | 1. Sample derived from participants in **clinical exercise trials.** 2. There was no true unexposed cohort. 3. There was no robust analytical control of confounders. 4. RTW based on **self-report (questionnaire)** and partly retrospectively reconstructed. 5. Inclusion only of those who have completed follow-up. |
| Starnoni (2018) | 1. Cohort was Single-center **and** selected. 2. No comparison cohorts not exposed. 3. Univariate analysis **only.** 4. Not clearly reported the rate of loss at follow-up. |
| Ullrich (2017) | 1. The cohort was selected. 2. No unexposed cohort. 3. Predominantly descriptive and univariate **analyses .** 4. Outcome based on **self-report** via questionnaire. |
| Ullrich (2018) | 1. The cohort was selected. 2. No unexposed cohort. 3. Outcome based on **self-report** via questionnaire. |
| Ullrich (2022) | 1. The cohort was selected. 2. No unexposed cohort. 3. Outcome based on **self-report** via questionnaire. |
| Yang (2021) | 1. No unexposed cohort. |
| Yang (2022) | 1. No unexposed cohort. |
| **Cross-sectional studies** | |
| Check (2019) | 1. Single-core sample. 2. Response rate **57%.** 3. No formal justification for sample size 4. Employment status detected via **self-report.** 5. **The application of Poisson's regression model is forced. In addition, The assumptions required by the model have not been reported.** |
| **Chen S. (2019)** | 1. Sample recruited in a single center, consecutive sampling. 2. No sample size calculation was reported. 3. RTW measured with **single self-report question.** |
| Chiu (2023) | 1. No formal justification of the sample size. 2. Outcome was collected via questionnarie (self-report). |
| Colombino (2020) | 1. Recruitment from **a single public cancer hospital.** 2. Outcome was collected via questionnarie (self-report). |
| Ito (2015) | 1. Low response rate (55.8%), possible selection bias. 2. No formal justification of the sample size. 3. **Low response rate.** 4. Data collected through **self-report questionnaires not clearly validated.** 5. Self-report outcome. |
| Kang (2022) | 1. No formal justification for sample size. 2. Response rate not reported. 3. Outcome collected through **interview (self-report).** |
| Lindbohm (2014) | 1. No formal justification for sample size. 2. Response rate not reported. 3. Outcome collected through **interview (self-report).** |
| Morales (2019) | 1. Recruitment from **a single public cancer.** 2. No formal justification of the sample size. 3. **Low response rate.** 4. Limited confounders control. 5. Outcome collected through **questionnaire (self-report).** |
| Paltrinieri (2020) | 1. **Low response rate.** 2. Not validated but described. 3. Outcome was self-report. |
| Rashid (2021) | 1. No justification of sample size. 2. Low response rate. 3. RTW outcome via **non-validated self-report.** 4. Outcome was self-report. |
| Ross (2012) | 1. No formal justification of the sample size. 2. Low response rate. 3. Instrument not validated. 4. Outcome was self-report. |
| Sesto (2022) | 1. Selected population. 2. No justification of sample size. 3. Low response rate. 4. Analysis limited to hypothesis testing. 5. many measures (RTW, needs) via adapted/non-validated questionnaire. |
| So (2019) | 1. Highly selected clinical sample. 2. No sample justification. 3. Low response rate. 4. Outcome was self-report. |
| Van Loon (2021) | 1. Non-representative clinical population. 2. No sample justification. 3. Moderate-low response rate. 4. self report outcome. 5. Limited confounders control. 6. ncomplete statistical analysis description. |
